# Multi-year analyses on three populations reveal the first stable QTLs for tolerance to rain-induced fruit cracking in sweet cherry (*Prunus avium* L.)

**DOI:** 10.1038/s41438-021-00571-6

**Published:** 2021-06-01

**Authors:** José Quero-García, Philippe Letourmy, José Antonio Campoy, Camille Branchereau, Svetoslav Malchev, Teresa Barreneche, Elisabeth Dirlewanger

**Affiliations:** 1 INRAE, Biologie du Fruit et Pathologie, Université de Bordeaux, UMR 1332, F-33140 Villenave d’Ornon, France; 2 CIRAD, UPR AIDA, University of Montpellier, TA B-115/02, Avenue Agropolis, 34398 Montpellier Cedex 5, France; 3 Department of Chromosome Biology, Max Planck Institute for Plant Breeding Research, Carl-von-Linné-Weg, 50289 Cologne, Germany; 4 Fruit Growing Institute – Plovdiv, 12 Ostromila Str., 4004 Plovdiv, Bulgaria

**Keywords:** Plant breeding, Plant genetics, Genetic markers

## Abstract

Rain-induced fruit cracking is a major problem in sweet cherry cultivation. Basic research has been conducted to disentangle the physiological and mechanistic bases of this complex phenomenon, whereas genetic studies have lagged behind. The objective of this work was to disentangle the genetic determinism of rain-induced fruit cracking. We hypothesized that a large genetic variation would be revealed, by visual field observations conducted on mapping populations derived from well-contrasted cultivars for cracking tolerance. Three populations were evaluated over 7–8 years by estimating the proportion of cracked fruits for each genotype at maturity, at three different areas of the sweet cherry fruit: pistillar end, stem end, and fruit side. An original approach was adopted to integrate, within simple linear models, covariates potentially related to cracking, such as rainfall accumulation before harvest, fruit weight, and firmness. We found the first stable quantitative trait loci (QTLs) for cherry fruit cracking, explaining percentages of phenotypic variance above 20%, for each of these three types of cracking tolerance, in different linkage groups, confirming the high complexity of this trait. For these and other QTLs, further analyses suggested the existence of at least two-linked QTLs in each linkage group, some of which showed confidence intervals close to 5 cM. These promising results open the possibility of developing marker-assisted selection strategies to select cracking-tolerant sweet cherry cultivars. Further studies are needed to confirm the stability of the reported QTLs over different genetic backgrounds and environments and to narrow down the QTL confidence intervals, allowing the exploration of underlying candidate genes.

## Introduction

Rain-induced fruit cracking has been traditionally considered as one of the most important agronomic problems in sweet cherry cultivation. Under unfavorable conditions, cracking can cause the loss of over 80% of the cherry (*Prunus avium* L.) harvest^1^. This phenomenon has been studied over one century and numerous reviews have been subsequently published^1–8^. It is a significant issue also in other fruits such as grapes, tomatoes, or plums^9^.

Fruit cracking is a highly complex phenomenon which has been related to a large number of factors, among which we may cite: cultivar, fruit size, cell size, firmness, skin characteristics, water uptake, osmolarity, rootstock, etc.^1,8^. The prevailing hypothesis used to explain rain-induced fruit cracking at a whole-fruit basis considers that water uptake increases fruit volume, surface area, and turgor, up to a point of critical turgor when skin ruptures and the fruit cracks^8^. However, the absence of significant turgor on sweet cherry fruits analyzed at maturity, as well as the fact that turgor did not respond to water uptake or transpiration^10^, casts serious doubts on this ‘traditional’ hypothesis, also referred to as the ‘critical turgor’ hypothesis. These conclusions were further supported by a study which proved that a sweet cherry fruit was far from behaving as an ideal osmometer^11^. In support of this result, a lack of a perfectly semipermeable skin was reported, the skin being permeable to solvent only, as well as to low-molecular solutes^12^; it was also proven that the flesh had a more negative water potential then the skin^13^. As an alternative, a different model was proposed in which cracking would be the result of a local defect, which in turn would provoke a zipper-type propagation in order to form a crack^8^. In line with this new ‘Zipper’ hypothesis/model, it was demonstrated that cell wall swelling, which favors fracture of epidermal cell walls, appears to be an early, critical, and essential step in a reaction of events, ultimately leading to cracking^14^. By using magnetic resonance imaging and optical coherence tomography, new evidence was provided that macrocracking resulted from a very localized water uptake through a microcrack, with its initial point the bursting of an individual outer-mesocarp cell^15^. Furthermore, by using light microscopy and the immunolabeling of cell walls, macrocrack propagation was related to cell death and to cell wall swelling^16^. Upon individual cells bursting, it was shown that turgor was removed, allowing in turn the cell wall swelling and provoking the release of malic acid, which caused a spread of damage^17^. Cell wall swelling was further directly related to a decrease in cell adhesion, affecting the structural backbone of the fruit (epidermis and hypodermis) and ultimately causing a crack^18^. Finally, it was demonstrated that cracking was primarily a function of wetness duration and the percentage of surface area wetted, rather than water uptake^19^. This finding was consistent with the Zipper model since the development of microcracks into macrocracks requires continuity of surface wetness on the fruit area^19^.

The existence of large differences in cracking tolerance/susceptibility between sweet cherry cultivars has been well documented^1,20,21^. While no strict resistance has yet been identified, tolerant cultivars exist, although these are unfortunately a minority. However, since the mechanism of cracking is still not completely understood, it has not yet been possible to design a reproducible and reliable test for the phenotyping of cultivar differences in terms of cracking susceptibility/tolerance. A laboratory-based assessment of cracking, initially proposed in the 1930s^22^, was further modified^23^. It basically consisted of the immersion of fruit, free of visual defects, in distilled water, followed by the counting, after 2, 4, and 6 h, of cracked fruits. A cracking index was established in order to consider the speed of cracking appearance and the overall proportion of cracked fruit. This test, also commonly called the Christensen test, has been thoroughly used to quantify the cracking susceptibility of the main sweet cherry commercial cultivars, but results obtained for the same cultivars at different sites were not consistently correlated^1^. In addition, the experience of numerous sweet cherry breeders suggests that the correlation between cracking susceptibility as measured by the Christensen test and as visually quantified in the field is often far from being highly significant^21^.

Although several agronomic methods have been proposed to prevent cracking, such as the use of physical barriers (rain covers), calcium salts or antitranspirants, and systems (helicopters or air blast blowers) to blow moisture off the fruit^8^, the use of tolerant cultivars remains the most economic and environment-friendly alternative. For this reason, breeding for cracking tolerance has always been considered a top priority^20,21,24,25^. However, no studies have previously been reported on the genetics of sweet cherry cracking tolerance. Among other fruit species, this type of work has solely been conducted in tomato^26–28^.

Quantitative trait locus (QTL) detection studies in sweet cherry have mostly dealt with phenology and fruit quality-related traits^29,30^. With the exception of fruit skin color, which is controlled by a single gene differentiating red skin and blushed skin cherries^31^, the other studied traits are highly polygenic. Major QTLs were detected for important agronomic traits such as bloom date and its components, chilling and heat requirements^32–34^, maturity date^35,36^, fruit weight (FW)^36–40^, fruit firmness (FF)^36,39–41^, and fruit sugar content and acidity^36^. Today, several sweet cherry breeding programs are routinely using molecular markers associated to key traits such as self-compatibility, fruit weight, fruit skin color, or maturity date, but no breeder has yet implemented marker-assisted selection for fruit cracking tolerance^30,42–44^. This approach would be particularly beneficial for sweet cherry breeders, given the difficulties to precisely evaluate fruit cracking tolerance within their plots.

The objectives of this study were the identification of sweet cherry rain-induced fruit cracking tolerance QTLs by analyzing three different mapping populations, as well as the evaluation of the potential of QTLs detected within future sweet cherry breeding programs oriented to the implementation of marker-assisted selection strategies.

## Results

### Phenotype variation

**Fig. 1:**
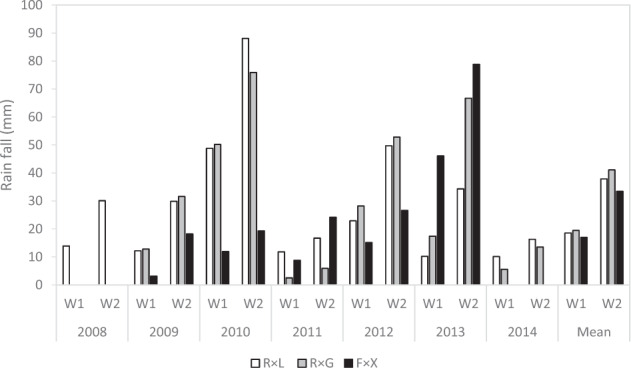
Genotypic differences in rainfall accumulation. Mean cumulated rainfall (in mm) per genotype within each population (R × L: ‘Regina’ × ‘Lapins; R × G: ‘Regina’ × ‘Garnet’; F × X: ‘Fercer’ × ‘X’) during one (W1) or two (W2) weeks before maturity

A high between-year variability was observed in terms of rainfall accumulated during the harvest period of the three populations studied. Years 2010, 2012, and 2013 showed high levels of cumulated rainfall for at least one or two populations, whereas others, such as 2011 or 2014, were characterized by rather low levels of accumulated rainfall (Fig. 1).

**Fig. 2:**
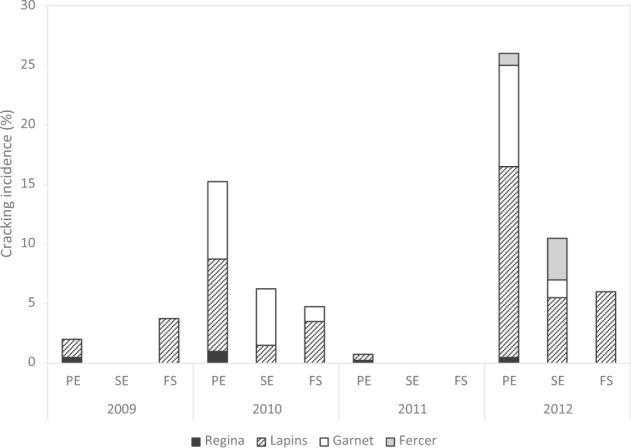
Cracking pattern variability among parents. Proportion of cracked fruits for each type of cracking (PE: pistillar end; SE: stem end; FS: fruit side) and the four known parents involved in the mapping populations (‘Regina’, ‘Lapins’, ‘Garnet’, and ‘Fercer’). In order to have a *y*-axis with a scale reaching a maximum of 100%, each individual parental cracking proportion was divided by four

The four parental genitors studied showed different levels and patterns of cracking incidence (Fig. 2). ‘Regina’ confirmed its tolerance to cracking, with only a very low level of cracking at the pistillar end (PE). No cracking was observed for ‘Fercer’ in 2011 but in 2012 it was visible for stem end (SE) and to a lesser extent, for PE cracking. ‘Lapins’ and ‘Garnet’ presented high levels of cracking susceptibility, in particular during the years 2010 and 2012. Cracking concerned mostly PE, but it was also observed for SE and fruit side (FS). Given the fact that the proportion of cracked fruits was based on the counting of 50 fruits, and that for numerous genotypes no cracked fruits were observed at a precise date, the distribution of cracking incidence was highly skewed towards 0 (Figs. S1–S3) and significantly far from normality, as confirmed by the results of Shapiro-Wilk tests (data not shown).

Significant differences in the distribution between years and also between types of cracking within each population were observed, years 2008, 2009, and 2011 were characterized by low mean values of cracking, with the exception of SE cracking for population F × X (Table 1). In contrast, during the years 2010, 2012, 2013, 2015, and 2016, higher mean values of cracked fruits were observed, reaching at least 40% for one specific family and type of cracking. Population R × L presented the lowest cracking mean values and was more sensitive to PE cracking. The same trend was observed for population R × G but with significantly higher mean values for all types of cracking. On the opposite, population F × X was characterized by SE cracking, with a relatively high global mean of 35% cracked fruit, followed by FS and PE cracking.

**Table 1:** Mean values of cracking proportion (number of cracked fruits per 50 observed fruits) for each year and each mapping population.

Trait	Year	Population		
		R × L	R × G	F × X
Pistillar end (PE)	2008	5.6	MD	MD
	2009	3.5	7.7	3.9
	2010	20.2	40.6	5.1
	2011	8.4	9.7	1.1
	2012	27.2	40.7	29.3
	2013	13.2	24.1	22.3
	2014	11.6	22.0	MD
	2015	21.3	39.0	9.2
	2016	MD	25.6	14.8
	Mean	13.9	26.2	11.8
Stem end (SE)	2008	1.2	MD	MD
	2009	0.5	3.3	20.6
	2010	6.8	15.4	34.1
	2011	2.6	2.8	26.0
	2012	10.5	33.4	48.2
	2013	3.5	12.0	46.6
	2014	2.4	6.9	MD
	2015	9.9	41.0	18.3
	2016	MD	22.3	51.6
	Mean	4.7	16.4	35.0
Fruit side (FS)	2008	4.5	MD	MD
	2009	0.4	1.2	5.4
	2010	6.0	14.5	9.0
	2011	1.5	1.6	1.6
	2012	23.3	29.4	33.3
	2013	3.4	14.9	41.1
	2014	1.9	10.3	MD
	2015	5.8	20.6	16.0
	2016	MD	25.0	24.1
	Mean	5.8	13.2	18.7

*R* × *L* ‘Regina’ × ‘Lapins’, *R* × *G* ‘Regina’ × ‘Garnet’, *F* × *X* ‘Fercer’ × ‘X’. *PE* pistillar end cracking, *SE* stem end cracking, *FS* fruit side cracking, *MD* missing data.

Within population R × L and for PE cracking, Spearman correlation coefficients were positive and significant for almost all two-year combinations, the highest being the correlation between 2014 and 2015 (0.66) (Table S1). For SE cracking, among the significant correlations, only one of them was above 0.5, between years 2010 and 2015, with a value of 0.61. Finally, a very low between-year stability was observed for FS cracking, since only three coefficients of correlation were significant and all had values below 0.3 (Table S1). A relatively similar situation was observed for population R × G. For PE cracking, the highest correlation coefficient was, as for population R × L, the one between 2014 and 2015 (0.65). For SE and FS cracking, 14 and 7 coefficients were significant but none had a value above 0.5 (Table S2). The lowest number of significant correlations was found within population F × X (Table S3).

Significant correlation coefficients between different types of cracking for each year of study were observed for all combinations although the highest were observed between PE and FS and between SE and FS cracking (Table 2). The highest values for population R × L were observed for the combinations PE-FS and SE-FS in 2012 (values of 0.62). For population R × G, two values of 0.67 were recorded for PE-FS and SE-FS correlations in 2016. Finally, a value of 0.71 was reached for population F × X in 2012 for the correlation between SE and FS cracking.

**Table 2:** Values of Spearman correlation coefficients between different types of cracking proportion (number of cracked fruits per 50 observed fruits) for each year of study and each population.

Population		Trait combination		
		PE–SE	PE–FS	SE–FS
R × L	2008	0.17	0.15	0.35^**^
	2009	0.26^**^	0.16	0.20^*^
	2010	0.31^**^	0.45^**^	0.54^**^
	2011	0.35^**^	0.56^**^	0.48^**^
	2012	0.46^**^	0.62^**^	0.62^**^
	2013	0.29^**^	0.18^*^	0.41^**^
	2014	0.18^*^	0.11	0.32^**^
	2015	0.18^*^	0.35^**^	0.45^**^
R × G	2009	0.46^**^	0.25^*^	0.38^**^
	2010	0.32^**^	0.66^**^	0.59^**^
	2011	0.13	0.02	0.30^**^
	2012	0.03	0.25^*^	0.36^**^
	2013	0.46^**^	0.58^**^	0.64^**^
	2014	0.33^**^	0.30^**^	0.40^**^
	2015	0.39^**^	0.45^**^	0.58^**^
	2016	0.54^**^	0.67^**^	0.67^**^
F × X	2009	−0.15	0.36^**^	0.02
	2010	0.47^**^	0.59^**^	0.38^**^
	2011	0.28^*^	0.24^*^	0.59^**^
	2012	0.36^**^	0.30^*^	0.71^**^
	2013	0.64^**^	0.58^**^	0.57^**^
	2015	0.51^**^	0.54^**^	0.54^**^
	2016	0.49^**^	0.67^**^	0.54^**^

Values above 0.5 are marked in bold.

*PE* pistillar end cracking, *SE* stem end cracking, *FS* fruit side cracking, *R* × *L* ‘Regina’ × ‘Lapins’, *R* × *G* ‘Regina’ × ‘Garnet’, *F* × *X* ‘Fercer’ × ‘X’. ^*^p-value < 0.05 and >0.01; ^**^p-value < 0.01.

For population R × L, FW was only significantly and positively correlated to SE and FS cracking, with maximum values of 0.36 (2008) and 0.37 (2012), respectively (Table S4). FF was not correlated with FS cracking, and only during 2 and 4 years with PE and SE cracking, respectively, with positive and low values, ranging between 0.21 and 0.33 (Table S4). The situation was relatively similar for population R × G, the maximum value being observed between FW and SE cracking in 2010 (0.38). In population F × X, two specificities were observed: first, PE cracking was significantly correlated to FW, with a maximum value of 0.49 in 2012; second, significant negative correlations were observed between FW or FF and all the cracking types, in particular during the year 2011, although absolute values were all below 0.4.

### Statistical modeling

The distribution of residuals illustrated the discrete nature of the studied variables. However, no heteroscedasticity was observed (results not shown). For population R × L (Table S5), 10, 5, and 5 models were tested for PE, SE, and FS cracking, respectively. For populations R × G and F × X (Tables S6 and S7), the numbers of models tested were 8, 5, 7 and 5, 7, 6, respectively. In the end, for each trait and population, the best models were selected: one with only rainfall-related covariates and one with rainfall- and fruit quality-related covariates (Tables 3 and 4). Differences were observed between cracking types for the same population. For instance, for population F × X, covariate WEEK1 was not significant for PE and FS cracking, whereas it was highly significant for SE cracking (Table 3). Between populations, a major difference was observed for covariates WEEK1 and WEEK2. Hence, their effect was not significant for PE and FS (with the exception of WEEK2) on population F × X, whereas it was highly significant for all types of cracking in populations R × L and R × G (Table 3). Concerning the covariate FF, it had significant effects on all types of cracking for populations R × L and R × G but only for SE cracking in the case of population F × X (Table 4). Covariate FW was significantly associated with all types of cracking in all populations, with the exception of FS and SE cracking in populations R × G and F × X, respectively (Table 4).

**Table 3:** Summary of selected ‘rainfall models’ (numbers correspond to highlighted models in Tables S5–S7) when considering only rainfall-related covariates: variance estimations (Year and Genotype) and *p*-value of *t*-tests for the significance of tested fixed effects.

			Studied variables											Adj. Stat.
Population	Model	Trait	Year (variance)	Genotype (variance)	DAY1	DAY2	DAY3	DAY4	DAY1–2	DAY1–3	DAY1–4	WEEK1	WEEK2	
R × L														
	6	PE	0.01354	0.03711		<0.0001	0.09					0.013	<0.0001	0.726
	3	SE	0.00469	0.00533	0.20					<0.0001		0.0001	0.06	0.543
	3	FS	0.01797	0.00239	0.28					<0.0001		<0.0001	0.005	0.591
R × G														
	5	PE	0.01412	0.04143	<0.0001	0.09						0.002	<0.0001	0.761
	3	SE	0.01785	0.00725	<0.0001	0.0006	0.09					0.01	<0.0001	0.675
	5	FS	0.02306	0.00479	<0.0001	0.002						<0.0001	<0.0001	0.717
F × X														
	3	PE	0.03095	0.01195	<0.0001	<0.0001	0.14	0.003						0.732
	5	SE	0.01043	0.02796					0.008			<0.0001		0.624
	2	FS	0.02991	0.00545	<0.0001	0.002	0.15	0.03					0.01	0.710

Adj. Stat.: conditional *R*^2^; DAY1, DAY2, DAY3, DAY4: amount of rainfall recorded 1, 2, 3, or 4 days before harvest; WEEK1, WEEK2: amount of rainfall cumulated during the week before or the two weeks before harvest. *R* × *L* ‘Regina’ × ‘Lapins’, *R* × *G* ‘Regina’ × ‘Garnet’, *F* × *X* ‘Fercer’ × ‘X’, *PE* pistillar end cracking, *SE* stem end cracking, *FS* fruit side cracking.

**Table 4:** Summary of selected ‘rainfall & fruit quality’ models (numbers correspond to highlighted models in Tables S5–S7) when considering rainfall- and fruit quality-related covariates: variance estimations (Year and Genotype) and *p*-value of *t*-tests for the significance of tested fixed effects.

			Studied variables													Adj. Stat.
Population	Model	Trait	Year (variance)	Genotype (variance)	DAY1	DAY2	DAY3	DAY4	DAY1–2	DAY1–3	DAY1–4	WEEK1	WEEK2	FF	FW	
R × L																
	10	PE	0.01262	0.03674		<0.0001	0.15					0.04	<0.0001	0.02	0.0006	0.736
	5	SE	0.00376	0.00363	0.11					<0.0001		0.001	0.08	<0.0001	<0.0001	0.555
	5	FS	0.01585	0.00142	0.23					<0.0001		<0.0001	0.006	<0.0001	<0.0001	0.600
R × G																
	7	PE	0.01572	0.04420	0.0002	0.23						0.001	<0.0001	0.06	<0.0001	0.773
	5	SE	0.01720	0.00656	<0.0001	0.004	0.10					0.004	<0.0001	<0.0001	<0.0001	0.690
	6	FS	0.02260	0.00436	<0.0001	0.005						<0.0001	<0.0001	<0.0001		0.719
F × X																
	5	PE	0.03493	0.01118	<0.0001	<0.0001	0.18	0.002							0.004	0.740
	6	SE	0.01687	0.02419					0.007			<0.0001		0.0002		0.637
	5	FS	0.03171	0.00419	0.0002	0.004	0.38	0.03					0.003		0.002	0.709

Adj. Stat.: conditional *R*^2^; DAY1, DAY2, DAY3, DAY4: amount of rainfall recorded 1, 2, 3, or 4 days before harvest; WEEK1, WEEK2: amount of rainfall cumulated during the week before or the two weeks before harvest. *R* × *L* ‘Regina’ × ‘Lapins’, *R* × *G* ‘Regina’ × ‘Garnet’, *F* × *X* ‘Fercer’ × ‘X’, *PE* pistillar end cracking, *SE* stem end cracking, *FS* fruit side cracking.

With model 0, by considering solely genotype and year effects, broad-sense heritabilities (H_BS_) in populations R × L and R × G, were the highest for PE cracking, followed by SE cracking with FS cracking being the least heritable (Table 5). For population F × X, SE cracking showed the highest H_BS_ value, slightly above the one estimated for PE cracking. The sharpest differences between traits were observed in population R × L, H_BS_ values ranging from 0.88 (PE) to 0.35 (FS). When introducing rainfall- and fruit quality-related covariates, results were relatively similar but H_BS_ values were always higher than those observed with model 0, with a few minor exceptions (SE and FS cracking for population R × L and model 1; FS cracking for population F × X and model 1).

**Table 5:** Broad-sense heritability (H_BS_) values estimated for each studied population and type of cracking with three models for variance estimation: (0) considering only genotype and year effects; (1) considering the same effects as model 0 plus rainfall-related and fruit-quality-related (FW and FF) covariates; (2) considering the same effects as model 0 plus rainfall-related covariates.

Population								
R × L			R × G			F × X		
PE	SE	FS	PE	SE	FS	PE	SE	FS
Model 0								
0.877	0.660	0.354	0.861	0.575	0.447	0.608	0.631	0.430
Model 1								
0.905	0.621	0.337	0.891	0.628	0.536	0.686	0.742	0.421
Model 2								
0.899	0694	0.451	0.877	0.640	0.557	0.695	0.723	0.452

*R* × *L* ‘Regina’ × ‘Lapins’, *R* × *G* ‘Regina’ × ‘Garnet’, *F* × *X* ‘Fercer’ × ‘X’, *PE* pistillar end cracking, *SE* stem end cracking, *FS* fruit side cracking.

### QTL detection

#### Single-QTL analyses

In terms of total number of QTLs, models 1 and 2 yielded, in most of the cases, a higher number than model 0, as summarized in Table 6.

**Table 6:** Synthesis of the number of QTLs detected per population and model at a single or multi-year basis for the three types of cracking tolerance.

Year	Pistillar end			Stem end			Fruit side		
	PE0	PE1	PE2	SE0	SE1	SE2	FS0	FS1	FS2
R × L									
2008	3	3	1	0	3	3	0	0	1
2009	1	2	2	2	3	1	2	1	1
2010	1	2	2	2	1	2	1	0	1
2011	1	4	5	1	1	2	0	1	1
2012	2	3	2	2	3	3	1	2	2
2013	1	1	3	3	3	5	0	0	3
2014	3	3	4	2	2	2	0	2	1
TOTAL	12	18	19	12	16	18	4	6	10
MY	8	8	9	6	9	10	3	7	6
R × G									
2009	2	2	2	0	3	1	1	2	2
2010	2	5	5	1	1	1	1	1	1
2011	5	4	5	1	0	0	1	3	3
2012	4	3	2	0	0	3	3	2	2
2013	3	2	2	2	3	3	1	4	4
2014	4	2	2	0	0	1	1	2	2
TOTAL	20	18	18	4	7	9	8	14	14
MY	11	9	9	5	8	8	4	4	5
F × X									
2009	1	2	5	4	3	0	4	0	0
2010	0	0	0	4	2	2	0	2	0
2011	0	0	1	1	2	4	0	3	3
2012	1	1	1	0	2	2	2	2	3
2013	0	0	0	0	1	1	2	1	0
TOTAL	2	3	7	9	10	9	8	8	6
MY	2	5	5	5	8	10	4	4	5

*PE0* pistillar end cracking, *SE0* stem end cracking, *FS0* fruit side cracking (model 0), *PE1* pistillar end cracking, *SE1* stem end cracking, *FS1* fruit side cracking (model 1), *PE2* pistillar end cracking, *SE2* stem end cracking, *FS2* fruit side cracking (model 2), *R* × *L* ‘Regina’ × ‘Lapins’, *R* × *G* ‘Regina’ × ‘Garnet’, *F* × *X* ‘Fercer’ × ‘X’, *MY* multi-year.

The results corresponding to model 1, which integrates the largest number of covariates, and hence the maximum level of available information, are presented and discussed (Tables S8–S10). Results for models 0 and 2 may be found in Tables S11–S16. For model 1, we will refer to PE1, SE1, and FS1 cracking (PE0, SE0, and FS0 cracking corresponding to model 0 and PE2, SE2, and FS2 cracking to model 2).

#### Population ‘Regina’ × ‘Lapins’ (R × L)

The use of predicted values allowed the detection of a higher number of QTLs for the three types of cracking (Table 6). This improvement was much more significant for SE and FS cracking, as compared to PE. Concerning PE1 cracking (Table S8), the QTL on linkage group (LG) R5 was the most stable, with values of phenotypic variance explained (PVE) ranging between 14.5% and 20%. QTLs on LGs R4 and R6 were also relatively stable, with 3 and 4 years of detection, and with maximum PVE values of 11.7% and 9.3%, respectively. QTLs mapped on the parent ‘Lapins’ were not detected for more than 1 year. For SE1 cracking (Table S8), only one QTL was detected consistently over a majority of years, on LG L4, and with PVE values comprised between 19.1% and 21.1%. The second most stable QTL was found on LG L2, detected during 3 years, and with a maximum PVE value of 15.3%. Two other QTLs, on LGs R3 and R6, were detected at least during 2 years, with PVE values reaching 14.7% and 13.4%, respectively. For the FS1 cracking, a QTL on LG R2 was detected during 3 years, with a maximum PVE value of 16.2% (Table S8).

**Fig. 3:**
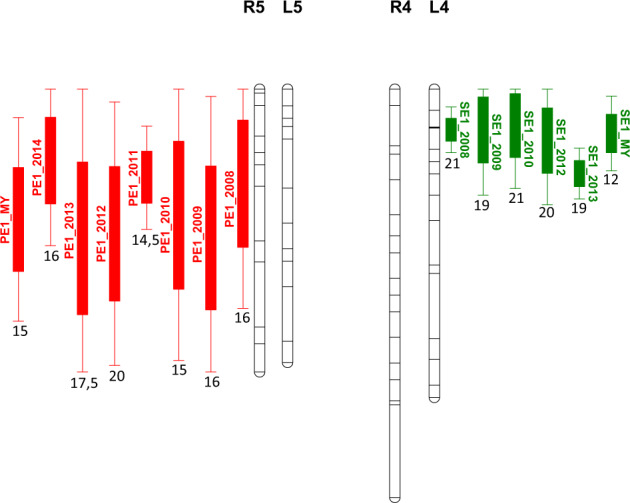
Graphical presentation of stable QTLs in population R × L. QTLs detected between 2008 and 2014, with the ‘one QTL per linkage group (LG)’ option, and both ‘single-year’ and ‘multi-year’ options of MultiQTL, within population R × L and model 1 (rainfall- and fruit quality-related covariates are considered). QTLs for pistillar end (PE1) cracking (in red) and stem end (SE1) cracking (in green) tolerance and for LGs R5 and L4, respectively, are presented. Mean values of phenotypic variance explained (PVE), expressed as a percentage, are indicated for each QTL. R: ‘Regina’; L: ‘Lapins’

Fig. S4 shows QTLs detected with multi-year analyses and for all traits. Despite the presence of relatively large confidence intervals, several QTL co-localizations for different types of cracking susceptibility could be hypothesized; it was the case for the three types of cracking on LG L2, and SE1 and FS1 cracking on LGs L4 and L7. The most stable QTLs, for PE1 cracking on LG R5 and for SE1 cracking on LG L4, which also showed the highest values of PVE, were not those detected with the shortest confidence intervals, 41.3 and 15.3 cM, respectively (Table S8). QTL positions for these two QTLs were not extremely stable between years, suggesting the existence of two (or more) putative QTLs in these LGs (Fig. 3). Hence, the PE1 QTL peak positions for LG R5 were, between years 2008 and 2014, at 19, 30, 26, 18, 29, 30, and 15 cM (Table S8). As for the SE1 QTL detected on LG L4, there was a high consistency in QTL positions between years 2008, 2009, 2010, and 2012, with peaks detected at 8, 8, 7, and 10 cM, respectively. However, in 2013, the peak was found at 17 cM and the confidence interval, between 12 and 22 cM, did not include the previous QTL peak positions detected between years 2008 and 2012 (Table S8).

#### Population ‘Regina’ × ‘Garnet’ (R × G)

A lower number of QTLs was detected for PE1 and PE2 models as compared to PE0 model, but the opposite was observed for SE and FS cracking (Table 6). As for population R × L, the largest and most stable QTL for PE1 cracking was found on LG R5, it was detected every year of the study, with PVE values ranging from 17.1% to 25.8% (Table S9). The second most important QTL was the one detected on LG R4, during 4 years, with a maximum PVE value of 17%. QTLs on LGs G2 and R6 were detected during 2 years but PVE values were always below 13%. No stable QTL was found for SE1 cracking. Only on LGs R3 and G8, QTLs were detected during 2 years (Table S9). For FS1 cracking, a QTL was detected on LG R2 during all years of study, with highly variable PVE values, ranging from 8.4% to 23.6%. A QTL was detected on LG G2 but only when using predicted values, both with FS1 (Table S9) and FS2 models (Table S15), during 5 years out of 6.

**Fig. 4:**
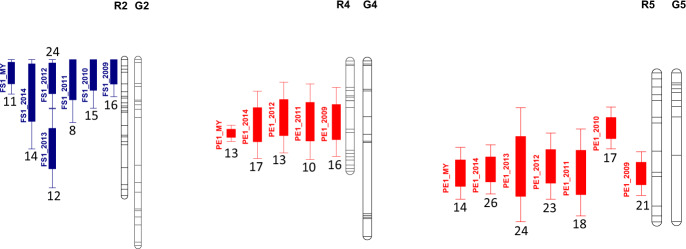
Graphical presentation of stable QTLs in population R × G. QTLs detected between 2009 and 2014, with the ‘one QTL per linkage group (LG)’ option, and both ‘single-year’ and ‘multi-year’ options of MultiQTL, within population R × G and model 1 (rainfall- and fruit quality-related covariates are considered). QTLs for pistillar end (PE1) cracking (in red) and fruit side (FS1) cracking (in blue) tolerance and for LGs R4, R5, and R2, respectively, are presented. Mean values of phenotypic variance explained (PVE), expressed as a percentage, are indicated for each QTL. R: ‘Regina’; G: ‘Garnet’

A lower number of co-localizations of QTLs for different types of cracking tolerance could be observed in population R × G, as compared to R × L (Fig. S5). Similarly to what was observed in population R × L, for the most important QTLs, in terms of stability and PVE, the ones for PE1 cracking on LG R5 and FS1 cracking on LG R2, the confidence intervals were quite large, 23 and 19 cM, respectively (Table S9). The existence of at least two-linked QTLs could be hypothesized for the PE1 QTL on LG R5 and the FS1 QTL on LG R2 (Fig. 4). For the former QTL, the peak positions were at 46, 25, 45, 42, 42, and 44 cM, from 2009 to 2014, with the year 2010 showing a significant difference (Table S9). In the case of the FS1 QTL on LG R2, consistent results were observed from 2009 to 2012 (peaks at 6, 7, 10, and 11 cM) but very different positions were recorded in 2013 and 2014, with peaks at 50 and 19 cM, respectively (Table S9). A higher consistency for the QTL peak position was observed for the PE1 cracking QTL on LG R4, with peaks at 33.2, 33, 30.8, and 34.8 cM for years 2009, 2011, 2012, and 2014, respectively (Table S9).

#### Population ‘Fercer’ × ‘X’ (F × X)

A higher number of QTLs was also detected by using predicted values for population F × X, specifically for PE and SE cracking (Table 6). Concerning PE1 cracking, very few QTLs were detected and all were mapped on the ‘X’ parent (Table S10). More QTLs were detected for SE1 cracking but without high between-year stability. Hence, only two QTLs were detected for a maximum of 2 years, on LGs F5 and X6 (Table S10). At a multi-year level, only QTLs detected on LGs F5, X4 and X6 explained more than 10% of the phenotypic variance (Table S10). For FS1 cracking, as for PE1 cracking, QTLs were only detected on parent ‘X’. The highest stability was observed for the QTL detected on LG X4, during 4 years, with PVE values ranging between 13.3% and 33.2% (Table S10).

Without considering QTLs with extremely large confidence intervals, the only clear co-localization between different types of cracking tolerance QTLs was found on LG X4, for SE1 and FS1 cracking (Fig. S6). As observed in populations R × L and R × G, the QTLs detected most frequently were not those showing the shortest confidence intervals on a multi-year basis. Hence, the FS1 QTL detected during 4 years on LG X4 had a confidence interval of more than 20 cM on a multi-year basis (Table S10).

#### Models comparison

In addition to the number of QTLs detected with the three different models, a major difference was observed for the QTL confidence intervals, which were almost systematically shorter when using predicted values from models 1 and 2 (Tables S8–S16). This improvement was observed for the three major QTLs detected for the three cracking traits (Fig. S7).

### Two-linked QTL analyses

**Fig. 5:**
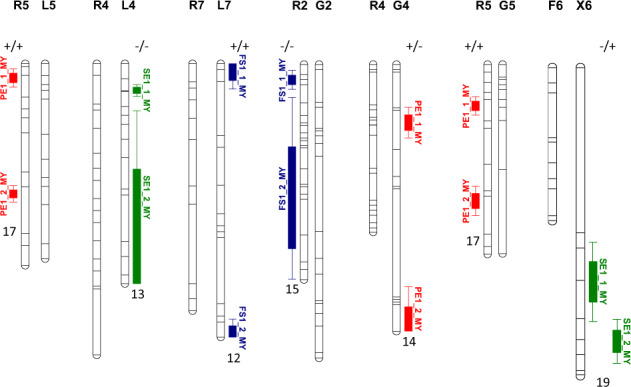
Graphical presentation of the main two-linked QTLs in all populations. Main QTLs detected with model 1 (rainfall- and fruit quality-related covariates are considered) and the ‘two-linked QTLs per linkage group (LG)’ and ‘multi-year’ options of MultiQTL, within populations R × L, R × G, and F × X, for pistillar end (PE1) cracking (in red), stem end (SE1) cracking (in green), and fruit side (FS1) cracking (in blue) tolerance. Mean values of phenotypic variance explained (PVE), expressed as a percentage, are indicated for each QTL pair. The sign of the effect of each QTL is also indicated: +/+ and −/− correspond to QTLs in coupling phase (the sign is arbitrarily assigned by MultiQTL to one of the two allelic forms) and +/− in repulsion phase. R: ‘Regina’; L: ‘Lapins’; G: ‘Garnet’; F: ‘Fercer’; X: ‘X’

The hypothesis of the existence of at least two QTLs on LG R5 for PE1 cracking was confirmed in populations R × L and R × G. With the multi-year option, the two QTLs had effects of the same sign, and were mapped in highly distant positions (Table 7 and Fig. 5). The peaks for both QTLs were mapped in the ‘Regina’ parent of each population in highly co-linear positions. The second two-linked QTL detection for PE1 cracking was observed on LG G4 during 2 years, 2009 and 2010. Both QTLs were mapped in clearly separate positions with the multi-year analyses and had opposite effects (Table 7 and Fig. 5).

**Table 7:** Synthesis of the most significant QTLs detected with the ‘two-linked QTL’ option of MultiQTL at a single and multi-year basis and with model 1 (considering rainfall- and fruit quality-related covariates).

Year	Model	LG	LOD	QTL1 peak	CI1 95% (cM)	QTL2 peak	CI2 95% (cM)	PVE (%)	*d*_1_	*d*_2_
R × L										
2009	PE1	R5	9.3	33.5	[2.4;57.5]	48.5	[37.3;57.5]	29.0	0.22	−0.09
2011	PE1	R5	7.4	34.3	[10.8;57.5]	47.0	[27.8;57.5]	32.5	0.30	−0.20
2014	PE1	R5	9.3	35.6	[10.1;57.5]	49.1	[36.8;57.5]	38.8	0.30	−0.17
MY	PE1	R5	79.9	4.0	[1.4;6.7]	37.0	[34.7;39.4]	17.4	0.08	0.12
2012	SE1	L4	11.3	8.2	[0.0;18.7]	25.8	[0.0;54.9]	22.1	−0.10	−0.04
MY	SE1	L4	61.6	7.6	[5.9;9.3]	47.1	[13.5;62.8]	17.0	−0.10	−0.01
2008	FS1	L7	6.1	3.2	[0.0;15.2]	66.4	[26.3;77.8]	23.5	0.07	0.04
2011	FS1	L7	7.2	5.8	[0.0;25.3]	47.2	[29.4;64.9]	23.5	0.08	−0.04
MY	FS1	L7	28.3	2.4	[0.0;7.2]	76.5	[72.7;77.8]	11.6	0.04	0.04
R × G										
2009	PE1	R5	12.1	16.2	[0.0;39.3]	48.4	[37.9;59.0]	23.6	0.10	0.15
2012	PE1	R5	12.7	13.2	[0.0;35.8]	48.4	[34.9;61.9]	26.9	0.10	0.14
2013	PE1	R5	14.3	13.0	[0.0;33.4]	52.6	[36.1;67.7]	33.6	0.15	0.22
MY	PE1	R5	66.9	14.8	[11.5;18.1]	48.8	[43.5;54.0]	17.3	0.11	0.11
2009	PE1	G4	6.2	21.8	[3.1;40.6]	75.2	[17.4;95.2]	25.3	0.23	−0.14
2010	PE1	G4	6.5	30.6	[0.0;68.5]	81.4	[27.0;95.2]	24.3	0.22	−0.13
MY	PE1	G4	29.0	20.8	[15.3;26.3]	93.9	[79.5;95.2]	13.7	0.15	−0.08
2011	FS1	R2	7.2	9.3	[0.0;34.4]	45.7	[6.7;76.7]	11.2	−0.10	−0.00
2012	FS1	R2	9.5	7.1	[0.0;22.5]	36.8	[0.0;76.7]	28.5	−0.10	−0.08
MY	FS1	R2	37.0	5.5	[2.1;8.9]	47.6	[11.8;76.7]	15.2	−0.10	−0.04
F × X										
2010	SE1	X6	15.1	64.8	[57.8;71.7]	85.1	[79.8;90.3]	48.8	−0.30	0.19
2012	SE1	X6	6.3	45.0	[10.9;79.2]	81.8	[56.9;94.1]	28.0	−0.20	−0.02
MY	SE1	X6	21.4	65.6	[53.5;77.7]	83.8	[77.2;90.5]	18.6	−0.16	0.09

*LG* linkage group, *LOD* logarithm of the odds ratio, *CI* confidence interval, *PVE* mean values of phenotypic variance explained (expressed as a percentage), *d*_*1*_ and *d*_*2*_ difference X(A) – X(B) according to the year of evaluation, where A and B are the two homozygotes at the marker loci; *R* × *L* ‘Regina’ × ‘Lapins’, *R* × *G* ‘Regina’ × ‘Garnet’, *F* × *X* ‘Fercer’ × ‘X’, *MY* multi-year, *PE1* pistillar end cracking, *SE1* stem end cracking, *FS1* fruit side cracking.

Concerning SE1 cracking, two-linked QTLs were detected in the population R × L on LG L4 only in 2012 (Table 7 and Fig. 5). On a multi-year level, the QTL detected on the upper part of the LG explained a larger part of the phenotypic variance (see comparison of *d*_1_ and *d*_2_ values, Table 7) and showed, accordingly, a shorter confidence interval (Table 7 and Fig. 5). Within population F × X, one region was considered, on LG X6. Two-linked QTLs were observed in 2010 and 2012, with opposite effects, mapped at 65 and 84 cM, with confidence intervals close to 15 cM for both QTLs, on a multi-year basis. (Table 7 and Fig. 5).

Finally, for FS1 cracking, two QTLs were detected in population R × L on LG L7 during 2008 and 2011. They were mapped with multi-year analyses at positions more than 70 cM apart, with effects of equal sign and confidence intervals with lengths less than 10 cM (Table 7 and Fig. 5). On population R × G, two-linked QTLs were mapped on LG R2 during 2 years, with effects of equal sign. The first QTL was mapped at the top of the LG with a confidence interval length less than 7 cM, whereas the second one was mapped at 47 cM and showed a much larger confidence interval. The effect of the allelic substitution (*d*) was higher as well for the QTL mapped on the upper part of the LG (Table 7 and Fig. 5).

#### Models comparison

The use of predicted values from models 1 and 2 significantly reduced confidence intervals for the most robust QTLs detected with the ‘2-QTL per LG’ option. Only the comparison between models 0 and 1 is presented in Fig. S8, showing a consistent improvement for the three major genomic regions.

## Discussion

This study highlighted the existence of stable QTLs for a highly complex trait, such as rain-induced fruit cracking. This was achieved by recording real-field data, by estimating the proportion of cracked fruit at harvest, and by differentiating three different fruit regions. Despite the risk of an important environmental component, due to the variability and unpredictability of rainfall events, the use of a high number of years and the integration of covariates related to rainfall accumulation, along with FW and FF, significantly improved the precision of the QTL detection analyses.

A high variability was observed in terms of cracking patterns both between mapping populations and between years of study (Table 1 and Fig. S1), confirming the high complexity of this trait. Within each population, one type of cracking was predominant, in agreement with the cracking patterns observed on parental cultivars (Fig. 2). Interestingly, ‘Lapins’ was more susceptible to cracking than ‘Garnet’, whereas, according to previous reports^20^, ‘Lapins’ has an intermediate level of cracking susceptibility while ‘Garnet’ is clearly described as susceptible. This reported behavior agrees with what was observed at a population level, since higher rates of cracking were recorded within population R × G, as compared to R × L (Table 1). Cultivar ‘Regina’ was relatively tolerant to cracking in our study, confirming previous results^21,45^. In all three populations, cracking was observed on several progenies even in the absence of significant cumulated rainfall before harvest. This cracking may be related to the fact that the plot where our populations were cultivated is fairly close to the Garonne River, with high levels of relative humidity, as well as a high frequency of morning dew. The occurrence of cracking in the absence of rain but in conditions of high relative humidity has been previously documented^46^.

In our study, correlations between FW and FF, and cracking incidence, were highly variable between years and populations, with several positive and significant values (Table S4). An increased cracking susceptibility was found in large-fruited cultivars^45,47^, whereas the same authors found no correlation between firmness and cracking. By evaluating cracking through the immersion test^23^, positive and significant correlations were found between FW and flesh firmness and rate of cracked fruits after 12 h of immersion, with mean values of 0.72 and 0.5, respectively^48^. In contrast, in a more recent study, no significant correlation was reported between cracking incidence as visually observed on harvested fruit and any fruit-related trait, fruit size, firmness, Brix value, or osmotic potential^49^. Overall, the relationship between FW and FF and cracking incidence remains largely dependent on the sample characteristics (number and type of cultivars), methodology used for cracking susceptibility estimation, and genotype × environment interactions.

The linear models used in our study were proved to be efficient in ‘correcting’ cracking proportion values recorded each year. From the comparison of models using only rainfall-related covariates and rainfall plus fruit quality-related covariates, it appears that rainfall parameters play a much more important role in explaining the genotypic differences within our mapping populations than traits, such as FW or FF, in terms of estimated heritability values (Table 5). In the three populations studied, FS cracking was the least heritable trait. A first explanation for this result may be that FS cracking is more complex than PE and SE cracking since in most cases, FS cracking is the result of an extension of a crack occurred in the PE or the SE regions^16^. This hypothesis was partially validated by the significant and relatively high correlation coefficients observed between both PE and FS cracking and SE and FS cracking in the three studied populations (Table 2). For a given individual, it was very rare to observe fruit with only FS cracking. Alternatively, it has been hypothesized that FS cracking might originate from water absorption through the vascular system, whereas PE and SE cracking would be essentially related to direct water absorption through the fruit surface, following a rain event^50^.

QTL detection analyses allowed us to identify numerous QTLs for the three types of cracking, confirming the complexity of this phenomenon. Furthermore, at least three highly stable QTLs were detected for each type of cracking, confirming preliminary results^35,51^. The fact that a higher number of QTLs was detected for PE and SE cracking, as compared to FS cracking, may be related, in addition to higher heritability estimates, to wetness duration, since a puddle of water forms in the SE cavity and a pending droplet remains attached at the PE, increasing the chances of cracking^8^. As for the higher heritability and higher number of QTLs detected for PE vs SE cracking, in particular within R × L and R × G populations, this could be due to the calcium gradient that has been observed within the fruit, with lower values recorded at the PE, and subsequently, higher cracking susceptibility^52^. During cracking, the loss of cell adhesion causes cell separation^14^ and the exposure of pectins on the fracture surface of a crack^16^. Since calcium is involved in the cross-linking of pectins, the lower calcium content in the PE of the fruit might be one of the reasons explaining the differences observed between PE and SE cracking. No major differences were observed in the most stable QTLs detected by using the three different models. However, confidence intervals were almost systematically shorter when using predicted values from models 1 and 2, which fully justifies the implementation of this approach (Tables S8–S16 and Figs. S7 and S8). Several interesting co-localizations of different types of cracking QTLs were observed (Figs. S2–S4). These co-localizations have to be considered cautiously since most of these QTLs were inconsistently detected, explained low levels of phenotypic variance, and were detected with large confidence intervals, even with the improvement allowed by the use of models 1 and 2. The complexity of the genetic determinism of cracking tolerance/susceptibility was further confirmed when conducting two-linked QTL analyses, since two putative QTLs were detected in five different LGs, including the three major QTLs previously mentioned (Table 7 and Fig. 5). In former studies conducted on populations R × L and R × G on other highly complex traits, such as FW and FF^39^, the presence of two QTLs per LG was also reported on almost all LGs when conducting multi-year analyses with MultiQTL software. Moreover, by using a different approach based on Bayesian statistics and multi-parental analyses, three QTLs for the trait FW were also detected on LG2^38^.

From a breeder’s perspective, the co-localization of QTLs of different agronomic traits is very important for the implementation of marker-assisted selection strategies. The existence of a QTL hotspot on LG2 was reported by working with a North American breeding germplasm population^53^. A high number of recombination events were identified within a region including QTLs for fruit size, fruit firmness, and bloom date, which had been reported in previous studies^33,37–39^. To these traits, we can now add cracking tolerance/susceptibility for all three types of cracking, although the most important and significant QTL on LG2 would concern FS cracking, detected on parent ‘Regina’. The LG4 also harbors highly important QTLs for key agronomical traits in sweet cherry and is considered to be a hotspot QTL LG^36,41^. Major QTLs have been identified for traits such as bloom date, maturity time, and fruit firmness^32,33,36,41^. In our study, relatively stable QTLs were detected for all types of cracking in this LG4.

Despite the use of predicted values and multi-year analyses, the smallest confidence intervals of the most significant QTLs (Table 7) covered 4–5 cM, which means at least 100 genes are present within these intervals (results not shown). Genes involved in the formation of cuticle^54,55^ or in the properties of cell walls^14,56^ might be potential candidate genes underlying the cracking tolerance/susceptibility QTLs. However, the potentially high number of genes involved in these important cell developmental processes impaired a realistic search of functional candidate genes within our confidence intervals, in contrast to other recent attempts dealing with traits such as bloom date^34,57^, FW^58^, or FF^39,41^. Fruit cracking in tomato is also a complex and polygenic trait and although robust QTLs were detected in at least five LGs, they explained relatively low percentages of phenotypic variance, always below 14%^26^. However, thanks to the use of a highly saturated map and a RIL population, these authors reported QTLs in narrow chromosomal intervals and for the main QTL on LG3, a promising marker within the sequence of a gene coding for an expansin was identified^26^. In contrast, none of the previously reported candidate genes potentially involved in tomato fruit cracking in transcriptomic studies, such as those involved in either cell wall^59^ or cuticle^60^ rearrangements during ripening, were located in any of the LGs with QTLs identified^26^.

In order to further validate and refine the confidence intervals of QTLs detected in this study, numerous possibilities could be explored. The two basic ways for improving QTL detection results could be summarized as follows: (i) increase the heritability of the trait under study, either by improving the protocols used for phenotyping or by reducing environmental variance in the experimental design; (ii) improve the detection power of the analysis, for which it has been widely demonstrated that increasing the number of analyzed genotypes is one of the most effective strategies. In the particular case of the study of cracking tolerance in sweet cherry, there seems to be a trade-off between these two strategies. While it is feasible to characterize cracking occurrence in a more detailed or precise way as the one we performed, this will be challenging to achieve if one intends to phenotype a very large number of progenies. In addition to classical biparental QTL detection, GWAS analyses can be an appealing strategy to explore a larger genetic diversity, although the need of phenotyping large numbers of genotypes will remain. Ideally, if the mechanistic basis of cracking was fully understood, it might be feasible to quantify physical and/or chemical parameters closely associated to cracking. Although the importance of the swelling of cell walls during cracking has been recently demonstrated, no significant correlation has been found between this feature and cracking susceptibility, within a panel of well-contrasted cultivars for this trait^56^.

From an applied breeding point of view, for the most significant QTLs highlighted in this study, diagnostic markers for marker-assisted selection might be developed and tested on a wider range of genetic backgrounds. In programs aiming at introgressing cracking tolerance from the cultivar ‘Regina’, diagnostic markers for the two PE cracking QTLs detected on LG R5 and for the QTL for FS cracking detected on the upper part of R2 (Fig. 5 and Table 7) could be particularly useful. Such type of diagnostic markers has been recently developed for other important agronomic traits such as FW or FF^30^.

## Conclusions

Our study provided the first detailed description of the genetic determinism of a highly complex trait in sweet cherry: rain-induced fruit cracking. As expected, the amount of rainfall prior to harvest was a major factor explaining the proportion of observed cracked fruits. However, significant levels of cracking were also reported during years with little rain. For the first time, cracking was decomposed into three main regions of the sweet cherry surface: PE, SE, and FS. The usefulness of this strategy was further confirmed by the identification of different QTLs underlying the variation of each of these three types of cracking, although several QTL co-localizations for different traits were also hypothesized. By working with three different mapping populations, at least one stable region was identified for each type of cracking, in three different LGs (2, 4, and 5), and explaining each a minimum of 20% of the phenotypic variance during 1 year of the study. Thanks to the availability of long series of data (between 7 and 8 years), we implemented simple linear models to predict cracking occurrence, by considering as covariates, different levels of cumulated rainfall before harvest, as well as two agronomically important traits in sweet cherry, such as FW and FF, previously reported as being correlated to cracking tolerance/susceptibility. These models allowed us to generate per year genotypic predictions of cracking incidence, which were subsequently used in QTL detection analyses, with a clear improvement in the precision of our QTL detection. Given the recent advances in the comprehension of the mechanistic bases of sweet cherry rain-induced fruit cracking, our study provides an important background for the identification of key genes involved in this phenomenon. Moreover, from an applied point of view, QTLs detected are sufficiently robust to envisage the implementation of marker-assisted selection strategies to develop high-quality and cracking-tolerant cultivars.

## Materials and methods

### Plant materials

Three segregating F_1_ adult sweet cherry populations were used in this study: (1) R × L, formed of 122 individuals derived from the cross between ‘Regina’ and ‘Lapins’ cultivars; (2) R × G, formed of 117 individuals derived from the cross between ‘Regina’ and ‘Garnet’ cultivars, and (3) F × X, formed of 67 individuals derived from a cross between cultivar ‘Fercer’ and an unknown parent called ‘X’. The parental cultivars represented a large range of cracking tolerance, from tolerant (‘Regina’), through intermediate (‘Lapins’), and susceptible (‘Garnet’ and ‘Fercer’), according to previous observations and breeders’ experience^21^. While the first population was planted in 2001, the two others were planted in 2002. Trees were cultivated at the Tree Experimental Unit (UEA) of the INRAE-Bordeaux research center, at Toulenne, located 50 km south-west from Bordeaux, France. Progenies were planted on their own roots, along with their grafted parents, in the same plot, on deep loamy soil on the bank of the Garonne River at 15 m above sea level (latitude 44.57 N, longitude 0.28 W). Distances between trees were of 2.5 m within rows and 5 m between rows. Weather conditions are characterized by a mild winter, relatively hot summers, and a yearly average rainfall of 825 mm. The plot studied was highly homogeneous in terms of soil composition and horticultural practices were consistently performed for the three populations. Artificial irrigation was provided to trees only during their juvenile period since sweet cherry trees planted on their own roots grow vigorously, soils are sufficiently rich, humid, and homogeneous in our experimental conditions and irrigation is not needed once trees have reached the adult phase.

### Trait measurement

The evaluation of rain-induced fruit cracking tolerance/susceptibility was already briefly described in preliminary studies^6,51^. Fruits were harvested when ripe, randomly from all tree areas, based on a subjective assessment of maturity, involving mainly firmness, texture, color, and taste. All fruit-quality measurements were conducted on the same day of harvest. From a batch of 50 fruits, every fruit was visually inspected and any observable crack was recorded, by differentiating between three distinct fruit regions: (i) the cracks at the stylar scar (also known as apical, pistillar end or nose cracks); (ii) the cracks at the stem cavity (also known as stem end or ring cracks); and (iii) the cracks at the cheek or suture side of the fruit (also known as fruit side cracks). For an individual fruit, one, two, or three types of cracking could co-exist. We considered the following terminology: pistillar end (PE), stem end (SE), and fruit side (FS) cracking. Although it has been recently confirmed that FS cracking is largely an extension of a crack that was initiated either at the PE or at the SE of the fruit^16^, every fruit that cracked at the FS was counted, independently of the cracking origin. The ‘geographical’ differentiation between the three areas considered was not always evident to establish, but if a PE or SE crack had just started to extend by 1 or 2 mm towards the FS, then this type of fruit was not counted as cracked at the FS. For the methodology on the phenotyping of fruit weight (FW) and firmness (FF), we may refer to^38,39,41^.

Phenotyping of cracking tolerance (PE, SE, and FS cracking), FW and FF was conducted on population R × L from 2008 till 2015 (8 years), on population R × G from 2009 till 2016 (8 years), and on population F × X from 2009 till 2013 and from 2015 till 2016 (7 years). Parental cultivars ‘Regina’ and ‘Lapins’ were evaluated from 2009 until 2012, whereas cultivars ‘Garnet’ and ‘Fercer’ were evaluated during 2010 and 2012 for ‘Garnet’ and 2011 and 2012 for ‘Fercer’.

### Statistical analyses

In order to illustrate the between-year variability in terms of rainfall accumulated during the harvest period, the mean amount of rainfall that all progenies (or hybrids) within each population received during the week or the two weeks before harvest, for the period 2008–2014, was calculated. Basic descriptive statistics were calculated for each trait, year, and population considered. Raw data were visualized through the use of box-plots. Cracking tolerance was computed with percentage of count data, a priori following a multinomial distribution. Hence, an *arcsin* (*rootsquare*) transformation was applied in order to stabilize the variance and to estimate genetic variances and heritabilities. We calculated Spearman coefficients of correlation (by using untransformed or raw data) in order to assess inter-year variation for each type of cracking tolerance and the relationships between the three types of cracking as well as cracking tolerance and other fruit quality-related traits, such as FW and FF.

In order to assess the effect of rainfall, FW, and FF on cracking tolerance, simple linear models were tested by using different covariates related to rainfall, as well as the values of FW and FF measured for each genotype. The linear models were tested with data produced from 2008 until 2014. From 2015 onwards, serious damage was observed on fruits, provoked by the invasive pest *Drosophila suzukii*^61^. The comparison of QTL results for cracking tolerance, produced with data collected before and after 2014, did not show significant differences. However, the evaluation of traits such as FW and more significantly, FF, became much more complicated, and hence these traits were not considered after 2014.

Meteorological data were extracted from a local weather station and only the rainfall occurring during a period of 14 days before harvest was deemed to have a putative influence on fruit cracking. Thus, for each genotype and year of study, the amount of rainfall recorded every day between 1 and 14 days before harvest was established. The amount of rainfall on the same day of harvest was not considered since fruits were harvested early in the morning and according to our experience, fruit will not crack (or not in a highly significant manner) a few minutes or hours after a rainfall event. All possibilities of cumulative rainfall were subsequently calculated, with intervals ranging from 2 to 14 days. In order to avoid the comparison of an extremely high number of models, 9 variables were arbitrarily chosen: the amount of rainfall recorded 1, 2, 3, or 4 days before harvest (called DAY1, DAY2, DAY3, and DAY4), the amount of rainfall cumulated between days 1 and 2 before harvest (DAY1–2), days 1 and 3 before harvest (DAY1–3), days 1 and 4 before harvest (DAY1–4), and the amount of rainfall cumulated during the week before harvest (WEEK1) or the two weeks before harvest (WEEK2).

These two types of models were adjusted:

Model ‘rainfall’:

$$Z_{ij} = \mu + \alpha _i + G_j + \mathop {\sum}\limits_{k = 1}^{k = K} {\left( {c_k \times R_{kij}} \right) + \varepsilon _{ij}}$$

Model ‘rainfall & fruit quality’:

$$Z_{ij} = \mu + \alpha _i + G_j + \mathop {\sum}\limits_{k = 1}^{k = K} {\left( {c_k \times R_{kij}} \right) + \mathop {\sum}\limits_{l = 1}^{l = L} {(d_l \times Q_{lij}) + \varepsilon _{ij}} }$$

where *Z*_*ij*_ was PE, SE, or FS cracking incidence, for year *i* and genotype *j*, *µ* was intercept, α_*i*_ a year *i* fixed effect, *G*_*j*_ a genotype *j* random effect, *R*_*k*_ a quantitative covariate *k* related to rainfall (correspondent parameter *c*_*k*_), *Q*_*l*_ a quantitative covariate *l* related to fruit quality (correspondent parameter *d*_*l*_), and ε_*ij*_ was a residual error.

Analyses were performed with the statistical SAS 9.3 software (SAS Institute, Cary, NC). In a first step (model ‘rainfall’), different combinations of rainfall-related covariates were included allowing for the integration of correlated but not collinear variables. For instance, DAY1 and DAY1–2 could be integrated into the model but not DAY1, DAY2, and DAY1–2, since DAY1–2 is equal to the sum of DAY1 and DAY2. Significance of fixed effects was tested through a type 3 test (*F* statistic). The strategy consisted of sequentially removing from the model those covariates that are without a significant effect on cracking in order to end up selecting the best model, in terms of values of adjustment statistics parameters, such as AIC (Akaike Information Criterion), AICC (corrected Akaike Information Criterion), and BIC (Bayesian Information Criterion). As these nested models differed in the number of fixed effects, but did not differ in their random effect, full maximum likelihood (ML) method was used to ensure valid comparisons. In a second step (model ‘rainfall & fruit quality’), once the most parsimonious set of rainfall-related covariates was selected, the covariates FF and FW were sequentially introduced into the model. For each population it was established, as before, through the analysis of adjustment statistics parameters, whether FF and/or FW contributed significantly to the cracking tolerance variation. In the end, by using restricted maximum likelihood method (REML), two models were selected for each type of cracking and population, one taking only rainfall-related covariates into account and the other one considering fruit quality-related covariates as well. The distribution of residuals was analyzed for the selected models by scatter plot between residuals and predicted values. For each model, we computed an adjustment statistic: a conditional *R*^2^, i.e., a squared correlation coefficient between observed values and predicted values, which incorporates best linear unbiased estimations of fixed effects and best linear unbiased predictions of random effect (genotype).

Finally, a measure of between-year stability, which can be viewed as a sort of broad-sense heritability (H_BS_), was estimated for each trait and population, as described in^32,33^. When considering only genotype and year effects, the VARCOMP procedure of SAS was used. Mean squares were estimated in order to calculate genetic variances. A second type of heritability was calculated by using the selected models with covariates. As the latter are quantitative and not categorical variables, VARCOMP procedure cannot be used and instead, we performed PROC MIXED. In this case, an estimation of the residual variance was generated and used for the heritability calculations. For each model, by using the OUTP command within the procedure MIXED, we generated predicted values for each genotype, conditional to the genotypes and years, considered as random effects. These approaches were, respectively, named ‘model 0’ (no covariates), ‘model 1’ (covariates rainfall and fruit-quality traits), and ‘model 2’ (covariate rainfall).

### QTL detection

Genetic maps of the three studied populations have been built after the genotyping of all individuals with the RosBREED Illumina Infinium cherry SNP array of 6 K SNP markers^62^. Details on the methodologies used for the maps’ construction of the populations R × L, R × G, and F × X can be found in^33,63^ and^41^, respectively. As sweet cherry is an allogamous and heterozygous species, the pseudo-testcross methodology^64^ was used to build separate parental maps. Overall, parental maps of ‘Regina’ and ‘Lapins’, for the population R × L, contained 136 and 127 SNP markers, respectively. ‘Regina’ and ‘Garnet’ parental maps, for the population R × G, were based on 142 and 137 SNP markers, respectively. Finally, the two parental maps of the population F × X consisted of 110 and 87 SNP markers, for ‘Fercer’ and ‘X’, respectively.

QTL detection and mapping were carried out using MultiQTL v2.6 software and the multiple interval mapping (MIM) approach (Haifa, Israel, 2005; http://www.multiQTL.com). Parameters for each QTL, namely LOD (logarithm of the odds ratio) values, position in cM, PVE, and the substitution effect (*d*^2^) were estimated according to^33^. Two types of analyses were performed. First, each year was analyzed independently for each trait. Second, the multi-environment option was used to combine all years of study for each trait and population, in order to increase the accuracy of the QTL detection. When conducting multi-year analyses, values of PVE and *d*^2^ were estimated for each year. However, for ease of reading, only the mean PVE and *d*^2^ values across years were presented to synthesize multi-year analyses.

For both ‘single’ and ‘multi-year’ analyses, the options of ‘one QTL per linkage group’ and ‘two-linked QTLs per linkage group’ were tested. When conducting analyses with the ‘two-linked QTLs per linkage group’ option, a LOD and a PVE value were calculated for each pair of detected QTLs. However, an estimation of the substitution effect was provided for each QTL (*d*_1_^2^ and *d*_2_^2^). As a much lower number of QTLs were detected with the ‘two-linked QTLs per linkage group’ option as compared to the ‘single QTL per linkage group’ option, only the most significant QTLs were presented. The choice of QTLs was based on three criteria: the detection of two-linked QTLs was significant at least during 1 year of the study and with a PVE value above 15%; the mean PVE value for the multi-year analyses reached 13%; the confidence intervals obtained with the multi-year analyses clearly separated both linked QTLs. The graphical presentation of linkage maps and QTLs was achieved using MAPCHART software version 2.2^65^.

QTL analyses were first performed on the measured variables of cracking tolerance. Subsequently, the same type of analysis was conducted after the *arcsin* (*rootsquare*) transformation of variables. No large differences were observed and only the results of analyses using transformed variables were presented. For each population and each year, three types of analyses were conducted: (i) by using the transformed observed phenotypic values; (ii) by using predicted values per genotype, provided by the selected model including rainfall- and fruit quality-related covariates; and (iii) by using predicted values per genotype, provided by the selected model including only rainfall-related covariates.

## Supplementary information

Table S1. Between-year values of Spearman correlation coefficients for cracking proportion (number of cracked fruits per 50 observed fruits) in population R×L. Values above 0.5 are marked in bold.

Table S2. Between-year values of Spearman correlation coefficients for cracking proportion (number of cracked fruits per 50 observed fruits) in population R×G. Values above 0.5 are marked in bold.

Table S3. Between-year values of Spearman correlation coefficients for cracking proportion (number of cracked fruits per 50 observed fruits) in population F×X.

Table S4. Values of Spearman correlation coefficients between fruit quality traits (fruit weight and fruit firmness) and cracking proportion (number of cracked fruits per 50 observed fruits) for each.

Table S5. List of covariates studied (marked with X) for each of the tested models for population R×L. Models selected (lowest values of AIC) are marked in bold.

Table S6. List of covariates studied (marked with X) for each of the tested models for population R×G. Models selected (lowest values of AIC) are marked in bold.

Table S7. List of covariates studied (marked with X) for each of the tested models for population F×X. Models selected (lowest values of AIC) are marked in bold.

Table S8. QTLs controlling cracking tolerance in population R×L for model 1, considering climatic (rainfall) and fruit quality-related (FW and FF) covariates. The option ‘1-QTL per linkage group’ was

Table S9. QTLs controlling cracking tolerance in population R×G for model 1, considering climatic (rainfall) and fruit quality-related (FW and FF) covariates. The option ‘1-QTL per linkage group’ was

Table S10. QTLs controlling cracking tolerance in population F×X for model 1, considering climatic (rainfall) and fruit quality-related (FW and FF) covariates. The option ‘1-QTL per linkage group’ was

Table S11. QTLs controlling cracking tolerance in population R×L for model 0, without covariates. The option ‘1-QTL per linkage group’ was used. Single and multi-year analyses are presented.

Table S12. QTLs controlling cracking tolerance in population R×G for model 0, without covariates. The option ‘1-QTL per linkage group’ was used. Single and multi-year analyses are presented.

Table S13. QTLs controlling cracking tolerance in population F×X for model 0, without covariates. The option ‘1-QTL per linkage group’ was used. Single and multi-year analyses are presented.

Table S14. QTLs controlling cracking tolerance in population R×L for model 2, considering climatic (rainfall) covariates. The option ‘1-QTL per linkage group’ was used. Single and multi-year analyses.

Table S15. QTLs controlling cracking tolerance in population R×G for model 2, considering climatic (rainfall) covariates. The option ‘1-QTL per linkage group’ was used. Single and multi-year analyses.

Table S16. QTLs controlling cracking tolerance in population F×X for model 2, considering climatic (rainfall) covariates. The option ‘1-QTL per linkage group’ was used. Single and multi-year analyses..

Fig. S1. Box-plots for the proportion of cracked fruits evaluated on population R × L from 2008 till 2014 by differentiating three types of cracking: pistillar end, stem end and fruit side.

Fig. S2. Box-plots for the proportion of cracked fruits evaluated on population R × G from 2009 till 2014 by differentiating three types of cracking: pistillar end, stem end and fruit side.

Fig. S3. Box-plots for the proportion of cracked fruits evaluated on population F × X from 2009 till 2013 by differentiating three types of cracking: pistillar end, stem end and fruit side.

Fig. S4. QTLs detected with the ‘multi-year’ option of MultiQTL within population R×L and model 1 (rainfall- and fruit quality-related covariates are considered) for pistillar end (PE1) cracking (in re

Fig. S5. QTLs detected with the ‘multi-year’ option of MultiQTL within population R×G and model 1 (rainfall- and fruit quality-related covariates are considered) for pistillar end (PE1) cracking (in re

Fig. S6. QTLs detected with the ‘multi-year’ option of MultiQTL within population F×X and model 1 (rainfall- and fruit quality-related covariates are considered) for pistillar end (PE1) cracking (in re

Fig. S7. Comparison of the major cracking tolerance QTLs detected with the ‘one QTL per linkage group’ and ‘multi-year’ options of MultiQTL and the three models considered: model 0 (no covariates), mo

Fig. S8. Comparison of the major cracking tolerance QTLs detected with the ‘two-linked QTLs per linkage group’ and ‘multi-year’ options of MultiQTL with two models considered: model 0 (no covariates)

Dataset phenotypic data.

## Data Availability

The datasets generated for this study, concerning all the genetic maps, can be found in the Genome Database for Rosaceae (https://www.rosaceae.org/publication_datasets). Accession no. tfGDR1019 and tfGDR1037. Concerning the raw phenotypic and climatic data (rainfall), all details can be found in the Excel file ‘Raw data’ within the supplementary information. Sheets ‘RxL’, ‘RxG’, and ‘FxX’ contain all phenotypic data from 2008 until 2016. Sheets ‘RxL model’, ‘RxG model’, and ‘FxX model’ contain all phenotypic and rainfall data from 2008 until 2014 used for the selection of linear models.
