# Supplementary material for: Multi-year analyses on three populations reveal the first stable QTLs for tolerance to rain-induced fruit cracking in sweet cherry (Prunus avium L.)
Source: Hortic Res. 2021 Jun 1;8:136. doi: 10.1038/s41438-021-00571-6 (PMC8166915; doi:10.1038/s41438-021-00571-6)
Supplement: Supplementary file 1 — Table S1. Between-year values of Spearman correlation coefficients for cracking proportion (number of cracked fruits per 50 observed fruits) in population R×L. Values above 0.5 are marked in bold. [file 41438_2021_571_MOESM1_ESM.docx]

**Table S1**. Between-year values of Spearman correlation coefficients for cracking incidence (number of cracked fruits per 50 observed fruits) in population R×L. Values above 0.5 are marked in bold.

|  | PE_09 | PE_10 | PE_11 | PE_12 | PE_13 | PE_14 | PE_15 | SE_09 | SE_10 | SE_11 | SE_12 | SE_13 | SE_14 | SE_15 | FS_09 | FS_10 | FS_11 | FS_12 | FS_13 | FS_14 | FS_15 |
| --- | --- | --- | --- | --- | --- | --- | --- | --- | --- | --- | --- | --- | --- | --- | --- | --- | --- | --- | --- | --- | --- |
| PE_08 | **0.52**** | **0.54**** | 0.46** | 0.29* | 0.37** | **0.50**** | 0.39** |  |  |  |  |  |  |  |  |  |  |  |  |  |  |
| PE_09 |  | **0.50**** | 0.30** | **0.58**** | **0.60**** | **0.53**** | **0.51**** |  |  |  |  |  |  |  |  |  |  |  |  |  |  |
| PE_10 |  |  | 0.37** | 0.44** | **0.55**** | **0.50**** | **0.52**** |  |  |  |  |  |  |  |  |  |  |  |  |  |  |
| PE_11 |  |  |  | 0.17 | 0.14 | 0.45** | 0.41** |  |  |  |  |  |  |  |  |  |  |  |  |  |  |
| PE_12 |  |  |  |  | **0.57**** | 0.43** | **0.54**** |  |  |  |  |  |  |  |  |  |  |  |  |  |  |
| PE_13 |  |  |  |  |  | **0.54**** | **0.54**** |  |  |  |  |  |  |  |  |  |  |  |  |  |  |
| PE_14 |  |  |  |  |  |  | **0.66**** |  |  |  |  |  |  |  |  |  |  |  |  |  |  |
| SE_08 |  |  |  |  |  |  |  | 0.24* | 0.34** | 0.22* | 0.04 | 0.15 | 0.02 | 0.13 |  |  |  |  |  |  |  |
| SE_09 |  |  |  |  |  |  |  |  | 0.49** | 0.01 | 0.47** | 0.26** | 0.14 | 0.35** |  |  |  |  |  |  |  |
| SE_10 |  |  |  |  |  |  |  |  |  | 0.03 | 0.36** | 0.42** | 0.19* | **0.61**** |  |  |  |  |  |  |  |
| SE_11 |  |  |  |  |  |  |  |  |  |  | -0.06 | 0.04 | 0.18 | 0.11 |  |  |  |  |  |  |  |
| SE_12 |  |  |  |  |  |  |  |  |  |  |  | 0.35** | 0.17 | 0.41** |  |  |  |  |  |  |  |
| SE_13 |  |  |  |  |  |  |  |  |  |  |  |  | 0.40** | 0.45** |  |  |  |  |  |  |  |
| SE_14 |  |  |  |  |  |  |  |  |  |  |  |  |  | 0.31** |  |  |  |  |  |  |  |
| FS_08 |  |  |  |  |  |  |  |  |  |  |  |  |  |  | 0.02 | 0.00 | 0.00 | 0.16 | 0.00 | 0.14 | 0.20 |
| FS_09 |  |  |  |  |  |  |  |  |  |  |  |  |  |  |  | 0.18 | -0.07 | 0.19 | -0.06 | 0.11 | 0.03 |
| FS_10 |  |  |  |  |  |  |  |  |  |  |  |  |  |  |  |  | -0.16 | 0.25* | 0.10 | 0.01 | 0.22* |
| FS_11 |  |  |  |  |  |  |  |  |  |  |  |  |  |  |  |  |  | -0.04 | -0.18 | -0.11 | 0.02 |
| FS_12 |  |  |  |  |  |  |  |  |  |  |  |  |  |  |  |  |  |  | 0.11 | 0.12 | 0.27* |
| FS_13 |  |  |  |  |  |  |  |  |  |  |  |  |  |  |  |  |  |  |  | 0.14 | 0.13 |
| FS_14 |  |  |  |  |  |  |  |  |  |  |  |  |  |  |  |  |  |  |  |  | 0.18 |

PE: pistillar end cracking; SE: stem end cracking; FS: fruit side cracking; * p-value <0.05 and >0.01; ** p-value <0.01.
