# Supplementary material for: Multi-year analyses on three populations reveal the first stable QTLs for tolerance to rain-induced fruit cracking in sweet cherry (Prunus avium L.)
Source: Hortic Res. 2021 Jun 1;8:136. doi: 10.1038/s41438-021-00571-6 (PMC8166915; doi:10.1038/s41438-021-00571-6)
Supplement: Supplementary file 2 — Table S2. Between-year values of Spearman correlation coefficients for cracking proportion (number of cracked fruits per 50 observed fruits) in population R×G. Values above 0.5 are marked in bold. [file 41438_2021_571_MOESM2_ESM.docx]

**Table S2**. Between-year values of Spearman correlation coefficients for cracking incidence (number of cracked fruits per 50 observed fruits) in population R×G. Values above 0.5 are marked in bold.

|  | PE_10 | PE_11 | PE_12 | PE_13 | PE_14 | PE_15 | PE_16 | SE_10 | SE_11 | SE_12 | SE_13 | SE_14 | SE_15 | SE_16 | FS_10 | FS_11 | FS_12 | FS_13 | FS_14 | FS_15 | FS_16 |
| --- | --- | --- | --- | --- | --- | --- | --- | --- | --- | --- | --- | --- | --- | --- | --- | --- | --- | --- | --- | --- | --- |
| PE_09 | **0.65**** | 0.40** | **0.59**** | 0.29** | **0.53**** | 0.48** | 0.32** |  |  |  |  |  |  |  |  |  |  |  |  |  |  |
| PE_10 |  | 0.46** | 0.41** | 0.32** | 0.49** | 0.42** | 0.19 |  |  |  |  |  |  |  |  |  |  |  |  |  |  |
| PE_11 |  |  | 0.48** | 0.33** | **0.52**** | **0.59**** | 0.33** |  |  |  |  |  |  |  |  |  |  |  |  |  |  |
| PE_12 |  |  |  | 0.37** | **0.51**** | 0.48** | 0.34** |  |  |  |  |  |  |  |  |  |  |  |  |  |  |
| PE_13 |  |  |  |  | **0.53**** | **0.53**** | 0.37** |  |  |  |  |  |  |  |  |  |  |  |  |  |  |
| PE_14 |  |  |  |  |  | **0.65**** | 0.39** |  |  |  |  |  |  |  |  |  |  |  |  |  |  |
| PE_15 |  |  |  |  |  |  | 0.44** |  |  |  |  |  |  |  |  |  |  |  |  |  |  |
| SE_09 |  |  |  |  |  |  |  | 0.25* | 0.12 | 0.37** | 0.00 | 0.24* | 0.15 | 0.18 |  |  |  |  |  |  |  |
| SE_10 |  |  |  |  |  |  |  |  | 0.05 | 0.37** | 0.21* | 0.12 | 0.46** | 0.17 |  |  |  |  |  |  |  |
| SE_11 |  |  |  |  |  |  |  |  |  | 0.00 | 0.00 | 0.14 | -0.04 | 0.12 |  |  |  |  |  |  |  |
| SE_12 |  |  |  |  |  |  |  |  |  |  | 0.26* | 0.10 | 0.44** | 0.41** |  |  |  |  |  |  |  |
| SE_13 |  |  |  |  |  |  |  |  |  |  |  | 0.33** | 0.25* | 0.19 |  |  |  |  |  |  |  |
| SE_14 |  |  |  |  |  |  |  |  |  |  |  |  | 0.38** | 0.31** |  |  |  |  |  |  |  |
| SE_15 |  |  |  |  |  |  |  |  |  |  |  |  |  | 0.34** |  |  |  |  |  |  |  |
| FS_09 |  |  |  |  |  |  |  |  |  |  |  |  |  |  | 0.16 | 0.07 | 0.15 | 0.17 | 0.15 | 0.08 | 0.14 |
| FS_10 |  |  |  |  |  |  |  |  |  |  |  |  |  |  |  | -0.10 | 0.26* | -0.08 | 0.01 | 0.23* | -0.04 |
| FS_11 |  |  |  |  |  |  |  |  |  |  |  |  |  |  |  |  | -0.15 | 0.05 | 0.10 | -0.10 | 0.03 |
| FS_12 |  |  |  |  |  |  |  |  |  |  |  |  |  |  |  |  |  | -0.07 | 0.21 | 0.25* | 0.42** |
| FS_13 |  |  |  |  |  |  |  |  |  |  |  |  |  |  |  |  |  |  | 0.20* | 0.20 | 0.18 |
| FS_14 |  |  |  |  |  |  |  |  |  |  |  |  |  |  |  |  |  |  |  | 0.12 | 0.29** |
| FS_15 |  |  |  |  |  |  |  |  |  |  |  |  |  |  |  |  |  |  |  |  | 0.35** |

PE: pistillar end cracking; SE: stem end cracking; FS: fruit side cracking; * p-value <0.05 and >0.01; ** p-value <0.01.
