# Supplementary material for: Multi-year analyses on three populations reveal the first stable QTLs for tolerance to rain-induced fruit cracking in sweet cherry (Prunus avium L.)
Source: Hortic Res. 2021 Jun 1;8:136. doi: 10.1038/s41438-021-00571-6 (PMC8166915; doi:10.1038/s41438-021-00571-6)
Supplement: Supplementary file 3 — Table S3. Between-year values of Spearman correlation coefficients for cracking proportion (number of cracked fruits per 50 observed fruits) in population F×X. [file 41438_2021_571_MOESM3_ESM.docx]

**Table S3**. Between-year values of Spearman correlation coefficients for cracking incidence (number of cracked fruits per 50 observed fruits) in population F×X.

|  | PE10 | PE11 | PE12 | PE13 | PE15 | PE16 | SE10 | SE11 | SE12 | SE13 | SE15 | SE16 | FS10 | FS11 | FS12 | FS13 | FS15 | FS16 |
| --- | --- | --- | --- | --- | --- | --- | --- | --- | --- | --- | --- | --- | --- | --- | --- | --- | --- | --- |
| PE09 | 0.39** | 0.40** | 0.36** | 0.32* | 0.30* | 0.40** |  |  |  |  |  |  |  |  |  |  |  |  |
| PE10 |  | 0.20 | 0.09 | 0.25* | 0.33** | 0.25 |  |  |  |  |  |  |  |  |  |  |  |  |
| PE11 |  |  | 0.15 | 0.29 | 0.02 | 0.26* |  |  |  |  |  |  |  |  |  |  |  |  |
| PE12 |  |  |  | 0.21 | 0.23 | 0.24 |  |  |  |  |  |  |  |  |  |  |  |  |
| PE13 |  |  |  |  | 0.15 | 0.23 |  |  |  |  |  |  |  |  |  |  |  |  |
| PE15 |  |  |  |  |  | 0.08 |  |  |  |  |  |  |  |  |  |  |  |  |
| SE09 |  |  |  |  |  |  | 0.47** | 0.31* | 0.14 | 0.40** | -0.04 | 0.30* |  |  |  |  |  |  |
| SE10 |  |  |  |  |  |  |  | 0.21 | 0.07 | 0.14 | 0.33** | 0.48** |  |  |  |  |  |  |
| SE11 |  |  |  |  |  |  |  |  | 0.05 | 0.13 | -0.15 | 0.31* |  |  |  |  |  |  |
| SE12 |  |  |  |  |  |  |  |  |  | 0.32* | -0.18 | -0.07 |  |  |  |  |  |  |
| SE13 |  |  |  |  |  |  |  |  |  |  | -0.11 | 0.14 |  |  |  |  |  |  |
| SE15 |  |  |  |  |  |  |  |  |  |  |  | 0.13 |  |  |  |  |  |  |
| FS09 |  |  |  |  |  |  |  |  |  |  |  |  | 0.18 | 0.29* | 0.06 | 0.28* | 0.17 | 0.09 |
| FS10 |  |  |  |  |  |  |  |  |  |  |  |  |  | 0.02 | 0.06 | 0.15 | 0.43** | 0.27* |
| FS11 |  |  |  |  |  |  |  |  |  |  |  |  |  |  | 0.02 | 0.07 | -0.14 | 0.02 |
| FS12 |  |  |  |  |  |  |  |  |  |  |  |  |  |  |  | 0.16 | 0.01 | 0.04 |
| FS13 |  |  |  |  |  |  |  |  |  |  |  |  |  |  |  |  | 0.30* | 0.16 |
| FS15 |  |  |  |  |  |  |  |  |  |  |  |  |  |  |  |  |  | 0.27* |

PE: pistillar end cracking; SE: stem end cracking; FS: fruit side cracking; * p-value <0.05 and >0.01; ** p-value <0.01.
