# Supplementary material for: Multi-year analyses on three populations reveal the first stable QTLs for tolerance to rain-induced fruit cracking in sweet cherry (Prunus avium L.)
Source: Hortic Res. 2021 Jun 1;8:136. doi: 10.1038/s41438-021-00571-6 (PMC8166915; doi:10.1038/s41438-021-00571-6)
Supplement: Supplementary file 4 — Table S4. Values of Spearman correlation coefficients between fruit quality traits (fruit weight and fruit firmness) and cracking proportion (number of cracked fruits per 50 observed fruits) for each. [file 41438_2021_571_MOESM4_ESM.docx]

**Table S4**. Values of Spearman correlation coefficients between fruit quality traits (fruit weight and fruit firmness) and cracking incidence (number of cracked fruits per 50 observed fruits) for each type of cracking, in each population and year of study (period 2008-2014).

| Population |  |  | Cracking type | | |
| --- | --- | --- | --- | --- | --- |
|  |  | Fruit quality traits | PE | SE | FS |
| R×L | 2008 | FW | -0.02 | 0.36** | 0.35** |
|  | 2009 | FW | 0.01 | 0.29** | 0.15 |
|  | 2010 | FW | -0.03 | 0.14 | 0.34** |
|  | 2011 | FW | 0.01 | 0.18 | 0.11 |
|  | 2012 | FW | 0.20 | 0.26* | 0.37** |
|  | 2013 | FW | 0.05 | 0.08 | 0.10 |
|  | 2014 | FW | 0.09 | 0.26** | 0.12 |
|  | 2008 | FF | 0.02 | -0.04 | -0.01 |
|  | 2009 | FF | 0.11 | -0.05 | -0.04 |
|  | 2010 | FF | 0.10 | 0.24* | 0.08 |
|  | 2011 | FF | 0.22* | 0.25* | 0.18 |
|  | 2012 | FF | 0.21* | 0.23* | 0.00 |
|  | 2013 | FF | 0.06 | 0.33** | 0.12 |
|  | 2014 | FF | 0.13 | 0.07 | 0.06 |
| R×G | 2009 | FW | 0.26* | 0.17 | 0.10 |
|  | 2010 | FW | 0.03 | 0.38** | 0.22* |
|  | 2011 | FW | 0.01 | 0.13 | -0.09 |
|  | 2012 | FW | -0.22 | 0.16 | 0.09 |
|  | 2013 | FW | -0.04 | 0.26** | 0.11 |
|  | 2014 | FW | 0.13 | 0.13 | 0.05 |
|  | 2009 | FF | 0.04 | 0.09 | 0.00 |
|  | 2010 | FF | 0.02 | 0.21* | 0.12 |
|  | 2011 | FF | 0.16 | 0.10 | 0.00 |
|  | 2012 | FF | 0.24* | 0.16 | 0.07 |
|  | 2013 | FF | 0.08 | 0.19* | 0.22* |
|  | 2014 | FF | 0.11 | 0.13 | 0.16 |
| F×X | 2009 | FW | 0.07 | 0.06 | 0.01 |
|  | 2010 | FW | -0.21 | 0.05 | -0.14 |
|  | 2011 | FW | -0.34 | -0.32** | -0.18 |
|  | 2012 | FW | 0.49** | 0.26 | 0.21 |
|  | 2013 | FW | 0.24* | 0.41** | 0.42** |
|  | 2009 | FF | -0.19 | -0.27* | -0.18 |
|  | 2010 | FF | -0.10 | -0.16 | 0.00 |
|  | 2011 | FF | -0.36** | -0.39** | -0.29* |
|  | 2012 | FF | 0.25 | 0.17 | 0.14 |
|  | 2013 | FF | -0.04 | -0.01 | 0.26* |

PE: pistillar end cracking; SE: stem end cracking; FS: fruit side cracking; FW: fruit weight; FF: fruit firmness; R×L: ‘Regina’ × ‘Lapins’; R×G: ‘Regina’ × ‘Garnet’; F×X: ‘Fercer’ × ‘X’; * p-value <0.05 and >0.01; ** p-value <0.01.
