# Supplementary material for: Multi-year analyses on three populations reveal the first stable QTLs for tolerance to rain-induced fruit cracking in sweet cherry (Prunus avium L.)
Source: Hortic Res. 2021 Jun 1;8:136. doi: 10.1038/s41438-021-00571-6 (PMC8166915; doi:10.1038/s41438-021-00571-6)
Supplement: Supplementary file 5 — Table S5. List of covariates studied (marked with X) for each of the tested models for population R×L. Models selected (lowest values of AIC) are marked in bold. [file 41438_2021_571_MOESM5_ESM.docx]

**Table S5**. List of co-variables studied (marked with X) for each of the tested models for population R×L. Models selected (lowest values of AIC) are marked in bold.

| Type of | Model | Co-variables | | | | | | | | | | | Adj. Stat. |
| --- | --- | --- | --- | --- | --- | --- | --- | --- | --- | --- | --- | --- | --- |
| cracking | sequence | DAY1 | DAY2 | DAY3 | DAY4 | DAY1-2 | DAY1-3 | DAY1-4 | WEEK1 | WEEK2 | FF | FW | AIC |
| PE | 1 | X |  |  |  | X | X | X | X | X |  |  | -255 |
| PE | 2 | X |  |  |  | X | X |  | X | X |  |  | -256 |
| PE | 3 | X |  | X |  | X |  |  | X | X |  |  | -256 |
| PE | 4 | X | X | X |  |  |  |  | X | X | X |  | -257 |
| PE | 5 | X | X | X |  |  |  |  | X | X |  |  | -256 |
| PE | **6** |  | X | X |  |  |  |  | X | X |  |  | -258 |
| PE | 7 |  | X | X |  |  |  |  | X | X | X |  | -259 |
| PE | 8 |  | X | X |  |  |  |  | X | X |  | X | -265 |
| PE | 9 | X | X | X |  |  |  |  | X | X |  | X | -263 |
| PE | **10** |  | X | X |  |  |  |  | X | X | X | X | -269 |
| SE | 1 | X |  |  |  | X | X | X | X | X |  |  | -832 |
| SE | 2 | X |  |  |  | X | X |  | X | X |  |  | -834 |
| SE | **3** | X |  |  |  |  | X |  | X | X |  |  | -836 |
| SE | 4 | X |  |  |  |  | X |  | X | X | X |  | -848 |
| SE | **5** | X |  |  |  |  | X |  | X | X | X | X | -888 |
| FS | 1 | X |  |  |  | X | X | X | X | X |  |  | -722 |
| FS | 2 | X |  |  |  | X | X |  | X | X |  |  | -724 |
| FS | **3** | X |  |  |  |  | X |  | X | X |  |  | -725 |
| FS | 4 | X |  |  |  |  | X |  | X | X | X |  | -729 |
| FS | **5** | X |  |  |  |  | X |  | X | X | X | X | -763 |

Adj. Stat. : adjustment statistics; DAY1, DAY2, DAY3, DAY4: amount of rainfall recorded one, two, three or four days before harvest; WEEK1, WEEK2: amount of rainfall cumulated during the week before or the two weeks before harvest; FF: fruit firmness; FW: fruit weight; AIC: Akaike information criterion; PE: pistillar end cracking; SE: stem end cracking; FS: fruit side cracking.
