# Supplementary material for: Multi-year analyses on three populations reveal the first stable QTLs for tolerance to rain-induced fruit cracking in sweet cherry (Prunus avium L.)
Source: Hortic Res. 2021 Jun 1;8:136. doi: 10.1038/s41438-021-00571-6 (PMC8166915; doi:10.1038/s41438-021-00571-6)
Supplement: Supplementary file 6 — Table S6. List of covariates studied (marked with X) for each of the tested models for population R×G. Models selected (lowest values of AIC) are marked in bold. [file 41438_2021_571_MOESM6_ESM.docx]

**Table S6**. List of co-variables studied (marked with X) for each of the tested models for population R×G. Models selected (lowest values of AIC) are marked in bold.

| Type of | Model | Co-variables | | | | | | | | | | | Adj. Stat. |
| --- | --- | --- | --- | --- | --- | --- | --- | --- | --- | --- | --- | --- | --- |
| cracking | sequence | DAY1 | DAY2 | DAY3 | DAY4 | DAY1-2 | DAY1-3 | DAY1-4 | WEEK1 | WEEK2 | FF | FW | AIC |
| PE | 1 | X |  |  |  | X | X | X | X | X |  |  | -75.2 |
| PE | 2 | X |  |  |  | X | X |  | X | X |  |  | -76.6 |
| PE | 3 | X |  | X |  | X |  |  | X | X |  |  | -76.6 |
| PE | 4 | X | X | X |  |  |  |  | X | X |  |  | -76.6 |
| PE | **5** | X | X |  |  |  |  |  | X | X |  |  | -77.2 |
| PE | 6 | X | X |  |  |  |  |  | X | X | X |  | -77.4 |
| PE | **7** | X | X |  |  |  |  |  | X | X | X | X | -92.1 |
| PE | 8 | X | X |  |  |  |  |  | X | X |  | X | -90.5 |
| SE | 1 | X |  |  |  | X | X | X | X | X |  |  | -429 |
| SE | 2 | X | X | X |  |  |  | X | X | X |  |  | -429 |
| SE | **3** | X | X | X |  |  |  |  | X | X |  |  | -428* |
| SE | 4 | X | X | X |  |  |  |  | X | X | X |  | -438 |
| SE | **5** | X | X | X |  |  |  |  | X | X | X | X | -462 |
| FS | 1 | X |  |  |  | X | X | X | X | X |  |  | -489 |
| FS | 2 | X |  |  |  | X | X |  | X | X |  |  | -490 |
| FS | 3 | X |  | X |  | X |  |  | X | X |  |  | -490 |
| FS | 4 | X | X | X |  |  |  |  | X | X |  |  | -490 |
| FS | **5** | X | X |  |  |  |  |  | X | X |  |  | -492 |
| FS | **6** | X | X |  |  |  |  |  | X | X | X |  | -505* |
| FS | 7 | X | X |  |  |  |  |  | X | X | X | X | -505 |

Adj. Stat. : adjustment statistics; DAY1, DAY2, DAY3, DAY4: amount of rainfall recorded one, two, three or four days before harvest; WEEK1, WEEK2: amount of rainfall cumulated during the week before or the two weeks before harvest; FF: fruit firmness; FW: fruit weight; AIC: Akaike information criterion; PE: pistillar end cracking; SE: stem end cracking; FS: fruit side cracking.
