# Supplementary material for: Multi-year analyses on three populations reveal the first stable QTLs for tolerance to rain-induced fruit cracking in sweet cherry (Prunus avium L.)
Source: Hortic Res. 2021 Jun 1;8:136. doi: 10.1038/s41438-021-00571-6 (PMC8166915; doi:10.1038/s41438-021-00571-6)
Supplement: Supplementary file 7 — Table S7. List of covariates studied (marked with X) for each of the tested models for population F×X. Models selected (lowest values of AIC) are marked in bold. [file 41438_2021_571_MOESM7_ESM.docx]

**Table S7**. List of co-variables studied (marked with X) for each of the tested models for population F×X. Models selected (lowest values of AIC) are marked in bold.

| Type of | Model | Co-variables | | | | | | | | | | | Adj. Stat. | |  |
| --- | --- | --- | --- | --- | --- | --- | --- | --- | --- | --- | --- | --- | --- | --- | --- |
| cracking | sequence | DAY1 | DAY2 | DAY3 | DAY4 | DAY1-2 | DAY1-3 | DAY1-4 | WEEK1 | WEEK 2 | FF | FW | | AIC | |
| PE | 1 | X | X | X | X |  |  |  | X | X |  |  | | -132 | |
| PE | 2 | X | X | X | X |  |  |  | X |  |  |  | | -134 | |
| PE | **3** | X | X | X | X |  |  |  |  |  |  |  | | -135 | |
| PE | 4 | X | X | X | X |  |  |  |  |  | X |  | | -134 | |
| PE | **5** | X | X | X | X |  |  |  |  |  |  | X | | -141 | |
| SE | 1 | X | X | X | X |  |  |  | X | X |  |  | | 78 | |
| SE | 2 | X | X | X | X |  |  |  | X |  |  |  | | 77 | |
| SE | 3 | X | X |  | X |  |  |  | X |  |  |  | | 75 | |
| SE | 4 | X | X |  |  |  |  |  | X |  |  |  | | 74 | |
| SE | **5** |  |  |  |  | X |  |  | X |  |  |  | | 73 | |
| SE | **6** |  |  |  |  | X |  |  | X |  | X |  | | 62 | |
| SE | 7 |  |  |  |  | X |  |  | X |  |  | X | | 72 | |
| FS | 1 | X | X | X | X |  |  |  | X | X |  |  | | -42 | |
| FS | **2** | X | X | X | X |  |  |  |  | X |  |  | | -44 | |
| FS | 3 | X | X | X | X |  |  |  |  |  |  |  | | -40 | |
| FS | 4 | X | X | X | X |  |  |  |  |  | X |  | | -38 | |
| FS | **5** | X | X | X | X |  |  |  |  | X |  | X | | -51 | |
| FS | 6 | X | X | X | X |  |  |  |  |  |  | X | | -44 | |

Adj. Stat. : adjustment statistics; DAY1, DAY2, DAY3, DAY4: amount of rainfall recorded one, two, three or four days before harvest; WEEK1, WEEK2: amount of rainfall cumulated during the week before or the two weeks before harvest; FF: fruit firmness; FW: fruit weight; AIC: Akaike information criterion; PE: pistillar end cracking; SE: stem end cracking; FS: fruit side cracking.
