# Supplementary material for: Multi-year analyses on three populations reveal the first stable QTLs for tolerance to rain-induced fruit cracking in sweet cherry (Prunus avium L.)
Source: Hortic Res. 2021 Jun 1;8:136. doi: 10.1038/s41438-021-00571-6 (PMC8166915; doi:10.1038/s41438-021-00571-6)
Supplement: Supplementary file 19 — Fig. S3. Box-plots for the proportion of cracked fruits evaluated on population F × X from 2009 till 2013 by differentiating three types of cracking: pistillar end, stem end and fruit side. [file 41438_2021_571_MOESM19_ESM.pdf]

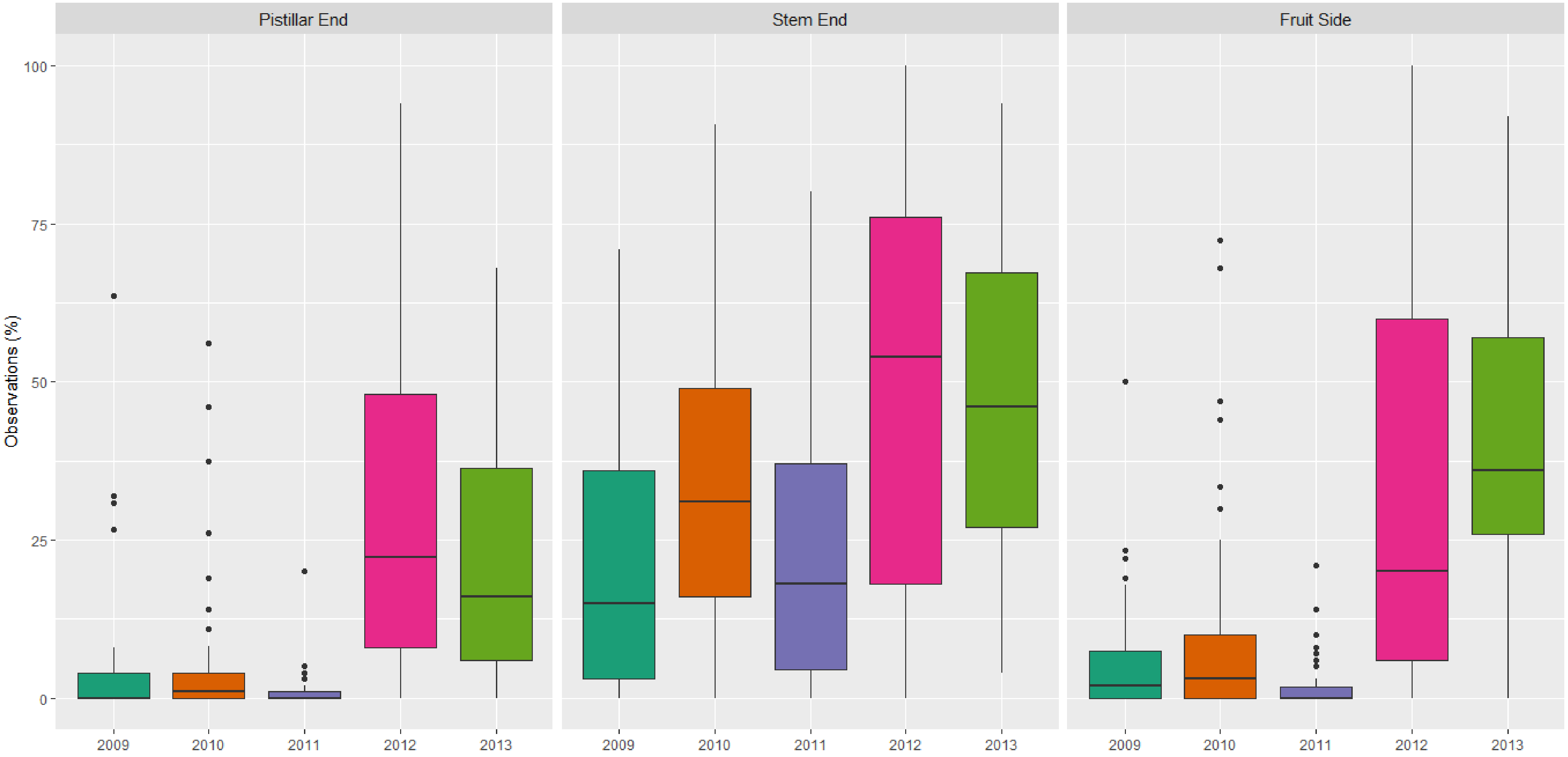

Figure S3. Box-plots for the proportion of cracked fruits evaluated on population F × X from 2009 till 2013 by differentiating three types of cracking: pistillar end, stem end and fruit side.
