# Supplementary material for: Multi-year analyses on three populations reveal the first stable QTLs for tolerance to rain-induced fruit cracking in sweet cherry (Prunus avium L.)
Source: Hortic Res. 2021 Jun 1;8:136. doi: 10.1038/s41438-021-00571-6 (PMC8166915; doi:10.1038/s41438-021-00571-6)
Supplement: Supplementary file 21 — Fig. S5. QTLs detected with the ‘multi-year’ option of MultiQTL within population R×G and model 1 (rainfall- and fruit quality-related covariates are considered) for pistillar end (PE1) cracking (in re [file 41438_2021_571_MOESM21_ESM.pdf]

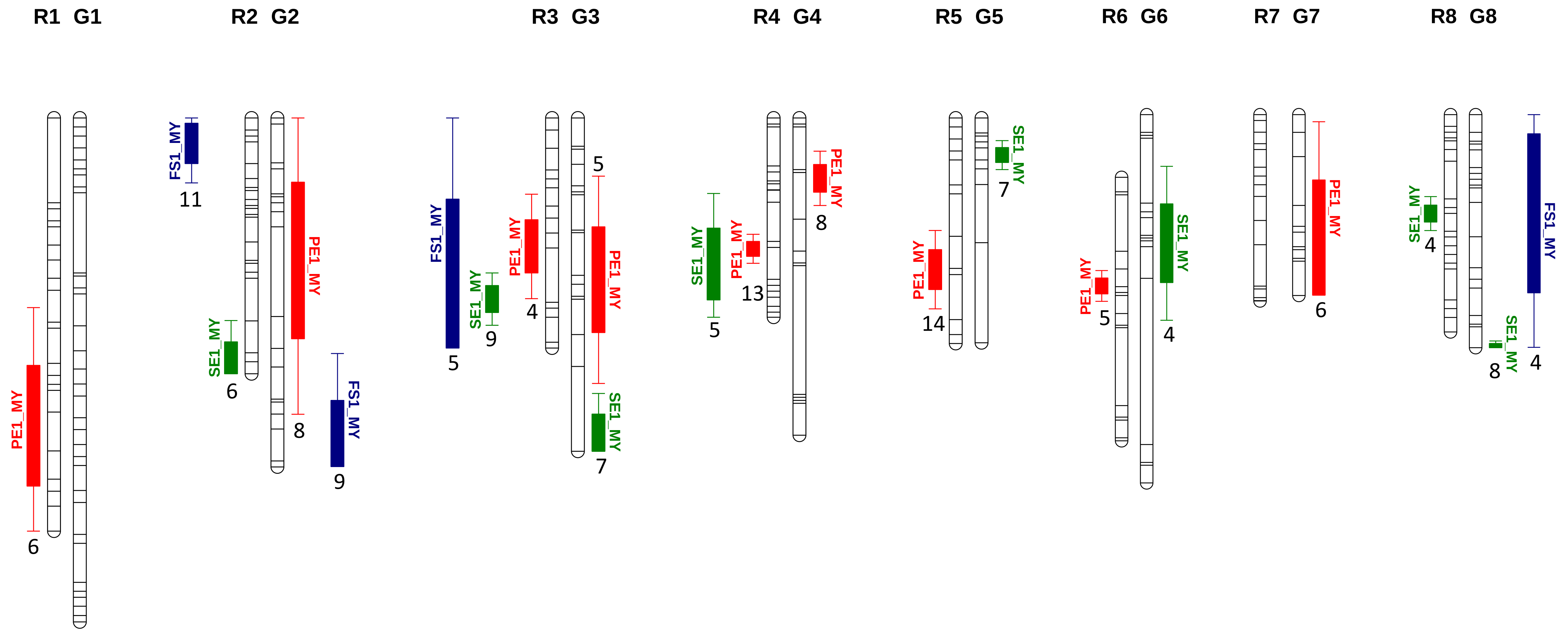

Figure S5. QTLs detected with the 'multi-year' option of MultiQTL within population R×G and model 1 (rainfall and fruit quality-related covariates are considered) for pistillar end (PE1) cracking (in red), stem end (SE1) cracking (in green) and fruit side (FS1) cracking (in blue) tolerance. Mean values of phenotypic variance explained (PVE), expressed as a percentage, are indicated for each QTL. R: 'Regina'; G: 'Garnet'.
