# Supplementary material for: Multi-year analyses on three populations reveal the first stable QTLs for tolerance to rain-induced fruit cracking in sweet cherry (Prunus avium L.)
Source: Hortic Res. 2021 Jun 1;8:136. doi: 10.1038/s41438-021-00571-6 (PMC8166915; doi:10.1038/s41438-021-00571-6)
Supplement: Supplementary file 23 — Fig. S7. Comparison of the major cracking tolerance QTLs detected with the ‘one QTL per linkage group’ and ‘multi-year’ options of MultiQTL and the three models considered: model 0 (no covariates), mo [file 41438_2021_571_MOESM23_ESM.pdf]

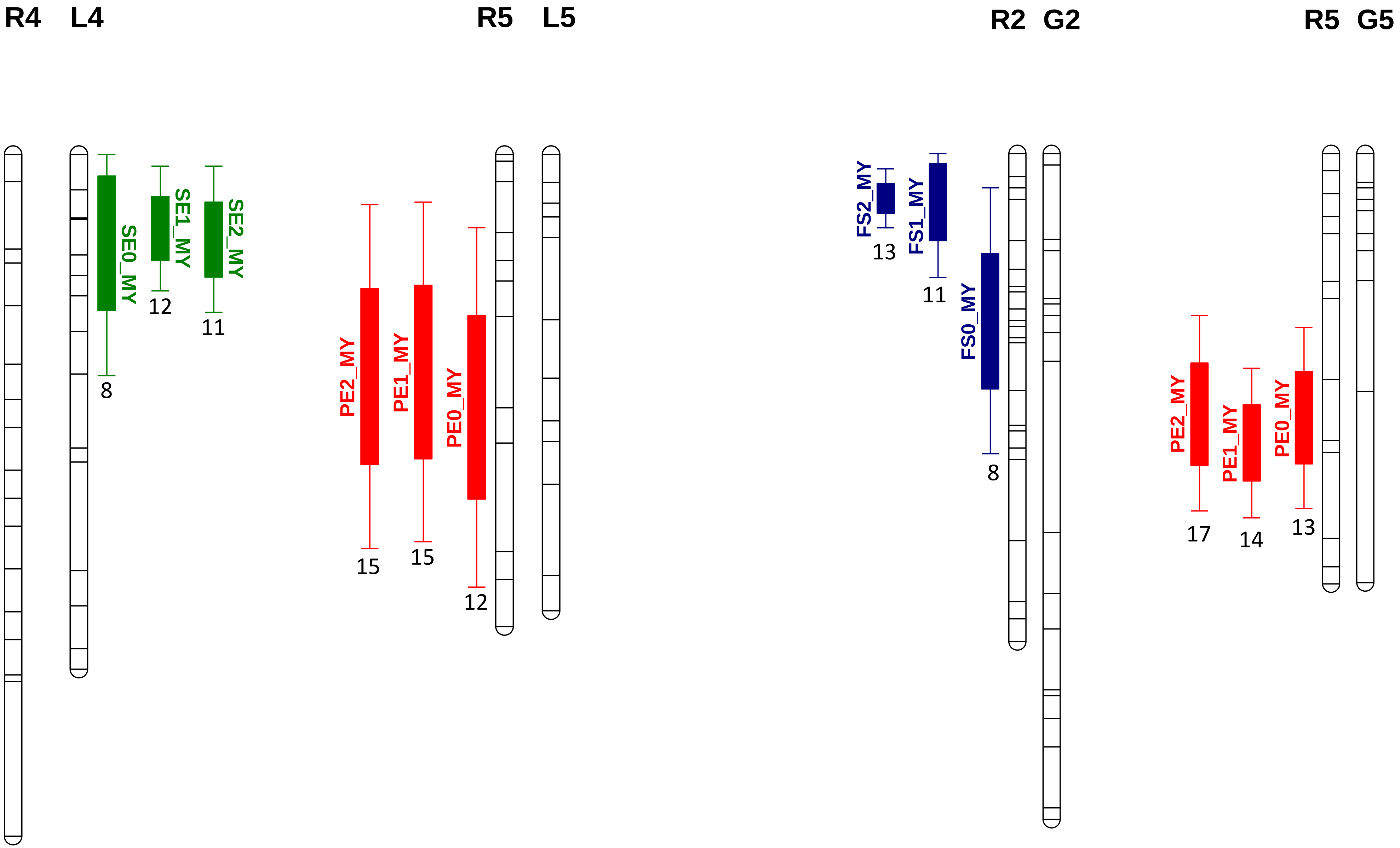

Figure S7. Comparison of the major cracking tolerance QTLs detected with the 'one QTL per linkage group' and 'multi-year' options of MultiQTL and the three models considered: model 0 (no covariates), model 1 (rainfall and fruit quality-related covariates) and model 2 (rainfall covariates) for pistillar end (PE0, PE1 and PE2) cracking (in red), stem end (SE0, SE1 and SE2) cracking (in green) and fruit side (FS0, FS1 and FS2) cracking (in blue). Mean values of phenotypic variance explained (PVE), expressed as a percentage, are indicated for each QTL. R: 'Regina'; L: 'Lapins'; G: 'Garnet'.
