# Supplementary material for: Multi-year analyses on three populations reveal the first stable QTLs for tolerance to rain-induced fruit cracking in sweet cherry (Prunus avium L.)
Source: Hortic Res. 2021 Jun 1;8:136. doi: 10.1038/s41438-021-00571-6 (PMC8166915; doi:10.1038/s41438-021-00571-6)
Supplement: Supplementary file 24 — Fig. S8. Comparison of the major cracking tolerance QTLs detected with the ‘two-linked QTLs per linkage group’ and ‘multi-year’ options of MultiQTL with two models considered: model 0 (no covariates) [file 41438_2021_571_MOESM24_ESM.pdf]

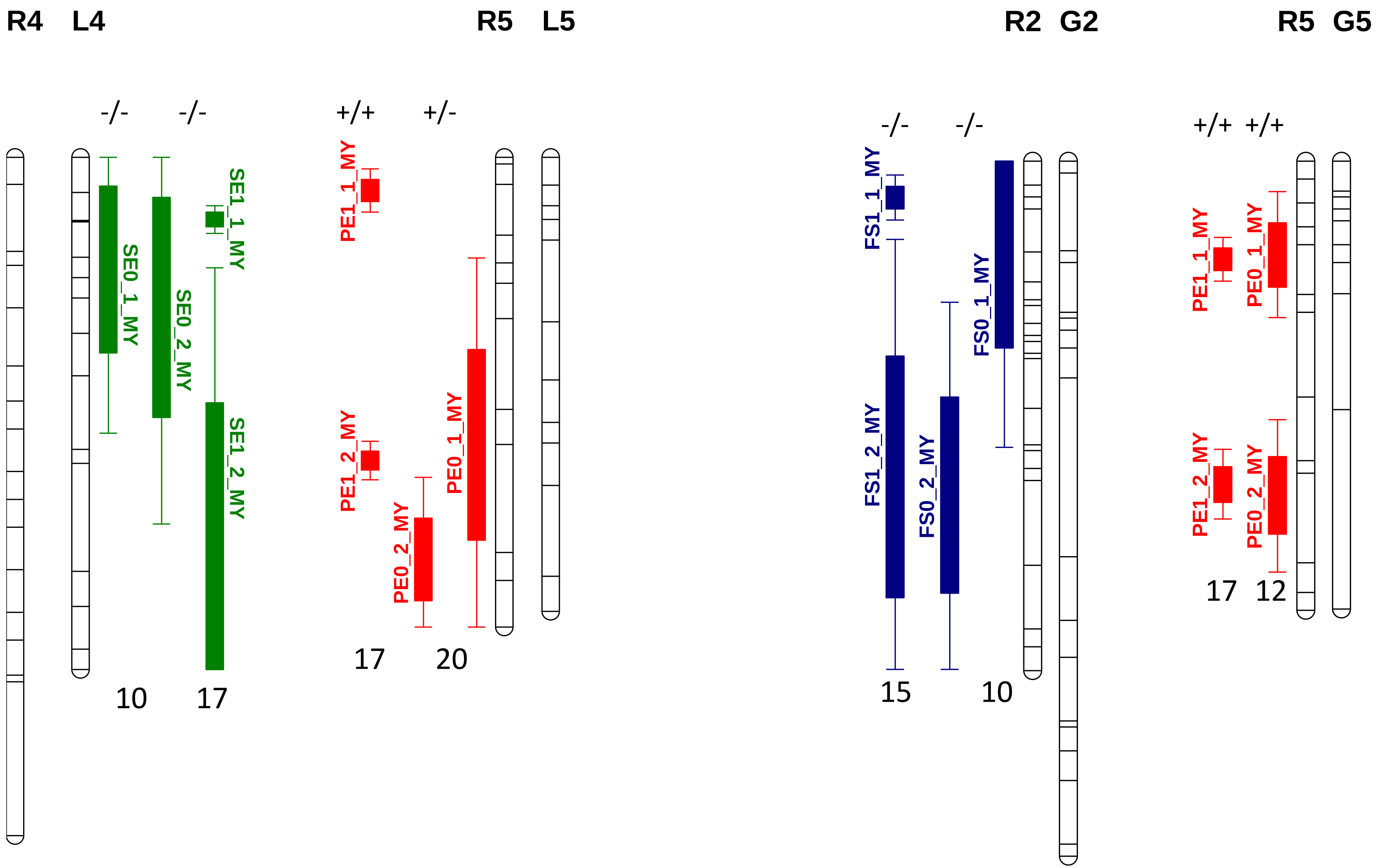

Figure S8. Comparison of the major cracking tolerance QTLs detected with the 'two-linked QTLs per linkage group' and 'multi-year' options of MultiQTL with two models considered: model 0 (no covariates) and model 1 (rainfall and fruit quality-related covariates) for pistillar end (PE0 and PE1) cracking (in red), stem end (SE0 and SE1) cracking (in green) and fruit side (FS0 and FS1) cracking (in blue). Mean values of phenotypic variance explained (PVE), expressed as a percentage, are indicated for each QTL pair. The sign of the effect of each QTL is also indicated: +/+ and -/- correspond to QTLs in coupling phase (the sign is arbitrarily assigned by MultiQTL to one of the two allelic forms) and +/- in repulsion phase. R: 'Regina'; L: 'Lapins'; G: 'Garnet'.
